# An Injectable Dual‐Function Hydrogel Protects Against Myocardial Ischemia/Reperfusion Injury by Modulating ROS/NO Disequilibrium

**DOI:** 10.1002/advs.202105408

**Published:** 2022-03-23

**Authors:** Tian Hao, Meng Qian, Yating Zhang, Qi Liu, Adam C. Midgley, Yangping Liu, Yongzhe Che, Jingli Hou, Qiang Zhao

**Affiliations:** ^1^ State key Laboratory of Medicinal Chemical Biology Haihe Laboratory of Sustainable Chemical Transformations Key Laboratory of Bioactive Materials (Ministry of Education) Frontiers Science Center for Cell Responses College of Life Sciences Nankai University Tianjin 300071 China; ^2^ School of Medicine Nankai University Tianjin 300071 China; ^3^ Tianjin Key Laboratory on Technologies Enabling Development of Clinical Therapeutics and Diagnostics School of Pharmacy Tianjin Medical University Tianjin 300070 China

**Keywords:** inflammation, ischemia/reperfusion injury, nitric oxide, oxidative stress, reactive oxygen species/nitric oxide equilibrium

## Abstract

Acute myocardial infarction (MI) is the leading cause of death worldwide. Exogenous delivery of nitric oxide (NO) to the infarcted myocardium has proven to be an effective strategy for treating MI due to the multiple physiological functions of NO. However, reperfusion of blood flow to the ischemic tissues is accompanied by the overproduction of toxic reactive oxygen species (ROS), which can further exacerbate tissue damage and compromise the therapeutic efficacy. Here, an injectable hydrogel is synthesized from the chitosan modified by boronate‐protected diazeniumdiolate (CS‐B‐NO) that can release NO in response to ROS stimulation and thereby modulate ROS/NO disequilibrium after ischemia/reperfusion (I/R) injury. Furthermore, administration of CS‐B‐NO efficiently attenuated cardiac damage and adverse cardiac remodeling, promoted repair of the heart, and ameliorated cardiac function, unlike a hydrogel that only released NO, in a mouse model of myocardial I/R injury. Mechanistically, regulation of the ROS/NO balance activated the antioxidant defense system and protected against oxidative stress induced by I/R injury via adaptive regulation of the Nrf2‐Keap1 pathway. Inflammation is then reduced by inhibition of the activation of NF‐*κ*B signaling. Collectively, these results show that this dual‐function hydrogel may be a promising candidate for the protection of tissues and organs after I/R injury.

## Graphical abstract

An injectable chitosan‐based hydrogel (CS‐B‐NO) is synthesized that could release nitric oxide (NO) in response to reactive oxygen species (ROS) stimulation and thereby modulate ROS/NO disequilibrium after ischemia/reperfusion (I/R) injury. Administration of CS‐B‐NO efficiently attenuates cardiac damage and adverse cardiac remodeling, promotes repair of the heart, and ameliorates cardiac function in a mouse model of myocardial I/R injury.

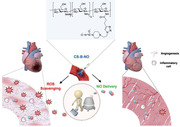

## 1 Introduction

Myocardial infarction (MI) is a common manifestation of ischemic heart disease and has become the leading cause of mortality worldwide.^[^ ^1^ ^]^ Coronary artery blockage often results in insufficient blood flow to the myocardium, leading to the apoptosis and necrosis of myocardial cells and thereby affecting cardiac function.^[^ ^2^ ^]^ Pathologically, MI is defined as long‐term ischemia resulting in myocardial cell necrosis. Since terminally differentiated cardiomyocytes are unable to regenerate, repair after MI often involves the formation of fibrotic scars, which are without the electrical conduction and contraction capabilities of cardiomyocytes, and this condition eventually evolves into heart failure, the leading cause of death.

Nitric oxide (NO), a multifunctional signaling molecule, plays a critical role in homeostatic regulation of the cardiovascular system.^[^ ^3^ ^]^ Many studies have shown the essential role of NO in angiogenesis and cardioprotection. Inhalation of NO can attenuate the adhesion and activation of leukocytes to the injured endothelium, thus reducing the overproduction of reactive oxygen species (ROS) in the heart.^[^ ^4^ ^]^ Accumulating evidence indicates that NO and its related signaling pathway are pivotal for the cardioprotective effect of preconditioning.^[^ ^5^ ^]^Therefore, different types of NO‐containing biomaterials (injectable hydrogels,^[^ ^6^ ^]^ electrospun cardiac patches, etc.) have been developed to realize the site‐specific delivery of NO with a controlled release profile,^[^ ^7^ ^]^ and some of them have shown therapeutic potential for the treatment of MI in both rodent and swine models.^[^ ^8^ ^]^

Myocardial ischemia/reperfusion (I/R) injury leads to oxidative stress that further impairs mitochondrial function. In the early stage of reperfusion, ADP is significantly reduced,^[^ ^9^ ^]^ and the reintroduction of O_2_ with reperfusion results in the overproduction of O_2_^•−^ and O_2_^•−^‐derived ROS in the mitochondria. O_2_^•−^ is readily converted to the more stable H_2_O_2_, which is an important member of ROS family as a physiological second messenger. However, the overproduction of H_2_O_2_ can induce oxidative stress and has been associated with the development and progression of cardiovascular disorders.^[^ ^10^ ^]^ In addition, myocardial ischemia and reperfusion serve as a stimulus that alters NO metabolism, which alters the balance between NO and O_2_^•−^ to favor oxidation reactions in the mitochondria rather than the normal physiological conditions (NO> O_2_^•−^).^[^ ^11^ ^]^

One way that ROS can affect NO responses is by oxidizing the sites in proteins with which NO reacts or sites that otherwise influence NO binding.^[^ ^11e^ ^]^ In addition, excess ROS can directly react with NO, which leads to the formation of peroxynitrite (ONOO^−^), a potent oxidant that mediates the oxidation of both nonprotein and protein thiols,^[^ ^12^ ^]^ and greatly increases related protein tyrosine nitration in the mitochondria.^[^ ^13^ ^]^ The nitration of tyrosine can deleteriously impact cellular function and viability because this specific modification is known to alter protein function in vitro.^[^ ^14^ ^]^

Complete NO signaling is essential for homeostasis in the cardiovascular system as it regulates vascular tone, platelet aggregation, and cardiac function. It was further proposed that the impairment of NO signaling leads to failure of the cardiovascular system, which is attributable to disequilibrium between ROS and RNS in the heart. In this regard, next‐generation NO delivery system should combine NO‐donation with other effects, especially ROS scavenging in order to enhance NO signaling.^[^ ^15^ ^]^

In this context, we designed a novel hydrogel, CS‐B‐NO, with dual functions: ROS scavenging and NO release. Specifically, boronate ester groups, which are efficiently and specifically cleaved upon ROS stimulation, were utilized as a protective group and conjugated with azide‐modified diazeniumdiolate to obtain a small‐molecule NO donor (B‐NO). This donor was then grafted onto the sidechain of natural chitosan by the click reaction to generate CS‐B‐NO. The CS‐B‐NO hydrogel demonstrated shear‐shinning properties; thus, its administration by intramyocardial injection could treat MI after myocardial I/R.^[^ ^16^ ^]^ The CS‐B‐NO hydrogel could modulate ROS/NO disequilibrium following I/R injury, thus inhibiting I/R‐induced oxidative stress and the inflammatory response via adaptive regulation of the nuclear factor‐erythroid 2‐related factor 2 (Nrf2)‐NF‐*κ*B defense system. Therefore, CS‐B‐NO exhibits enhanced therapeutic efficacy over that of hydrogels with the ability to only release NO or scavenge ROS.

## 2 Results

### 2.1 Fabrication and Characterization of an ROS‐Responsive, NO‐Releasing Hydrogel

I/R cardiac injury is often characterized by a burst of ROS, which exacerbates ischemic damage. Inspired by this pathological feature, we designed a microenvironment‐responsive hydrogel that could release NO upon ROS stimulation. CS‐B‐NO was synthesized through two steps.^[^ ^6^ ^]^ First, alkyne‐substituted chitosan (alkynyl‐CS) was synthesized through the reaction between 4‐pentynoic acid and chitosan according to a reported method.^[^ ^17^ ^]^ Then, a small‐molecule NO donor (B‐NO), which contained an azide group at its end, was grafted onto the alkynyl‐CS by the click reaction. The chemical structure of CS‐B‐NO was confirmed by ^1^H NMR (Figures S1 and S2, Supporting Information).

**Figure 1:**
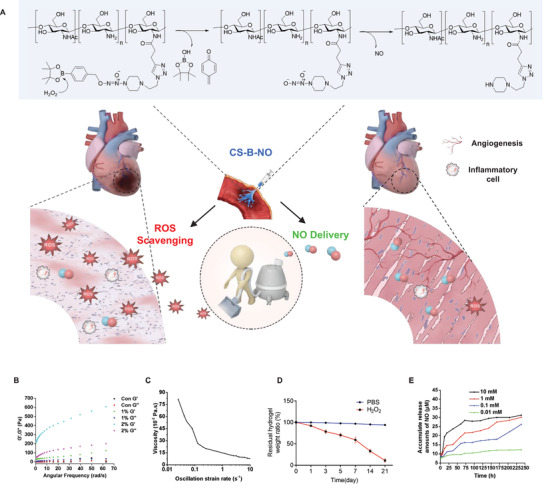
Preparation and characterization of the injectable chitosan hydrogel with ROS‐responsive NO‐releasing function (CS‐B‐NO). A) Schematic illustration of the treatment of I/R heart injury by CS‐B‐NO. B) Rheology properties of CS‐B‐NO was evaluated by frequency sweep experiments at a constant strain of 1%. C) Oscillatory strain sweeps of CS‐B‐NO. D) In vitro degradation of CS‐B‐NO hydrogel in the presence or absence of H_2_O_2_ (100 µm), and the residual weight of dried hydrogel was measured at different intervals. Data were presented as mean ± SEM (n = 3 individual experiments). E) In vitro generation of NO from the CS‐B‐NO hydrogel (5mg) in 5 mL of PBS buffer (pH 7.4) with hydrogen peroxide of various concentrations. The cumulative releasing amount of NO was calculated. Data were presented as mean ± SEM (n = 3 individual experiments).

Rheological measurements (**Figure** **1**B) of the storage and loss moduli demonstrated that CS‐B‐NO formed a robust hydrogel due to dynamic boronate ester bonding between phenylboronate ester and hydroxyl/amino on chitosan.^[^ ^18^ ^]^ With increasing CS‐B‐NO concentration from 1 to 2 wt.%, the mean G' value evidently increased from 100 to 608 Pa. The formed CS‐B‐NO hydrogel was stable when subjected to an inversion test. In particular, the CS‐B‐NO hydrogel exhibited shear‐thinning behavior and could transform to liquid‐like flow under high strain. As a result, measurement of dynamic viscosity demonstrated a decrease in viscosity with increasing strain rate, reflecting shear thinning properties that are characteristic of shear‐rate‐dependent breakage of interchain linkages in hydrogels (Figure 1C). Besides, the CS‐B‐NO hydrogel was easily injectable and readily re‐formed after injection into the targeted location, such as cardiac tissue. In vitro degradation profile of the hydrogel was evaluated, and the results showed that CS‐B‐NO hydrogel was relatively stable in PBS without detectable weight loss. The degradation was accelerated by the addition of H_2_O_2_ that mimics the situation of ROS burst after I/R injury; the CS‐B‐NO hydrogel experienced weight loss of more than 90% after 21 days of incubation (Figure 1D).

The in vitro NO‐release behavior of CS‐B‐NO was evaluated using a Griess assay. As shown by the results, no released NO was detected in the absence of H_2_O_2_. After incubation with H_2_O_2_ of various concentrations (10 mm, 1 mm, 100 µm, or 10 µm), the absorbance at 540 nm (maximal absorption wavelength) increased as the reaction progressed, reflecting the controlled and sustained release of NO from CS‐B‐NO (Figure 1E).

Meanwhile, a CS‐B hydrogel that can respond to H_2_O_2_ and produce fluorescence was synthesized as a control (Figures S3–S8, Supporting Information). To investigate the sensitivity of CS‐B to H_2_O_2_, the fluorescence response of CS‐B towards H_2_O_2_ was evaluated with the excitation wavelength *λ* = 680 nm. CS‐B showed very weak fluorescence at 720 nm (*λ* = 680 nm), but the addition of H_2_O_2_ led to a gradual increase in fluorescence with reaction time (Figure S9, Supporting Information).

### 2.2 The CS‐B‐NO Hydrogel Modulated ROS/NO Disequilibrium In Vitro

**Figure 2:**
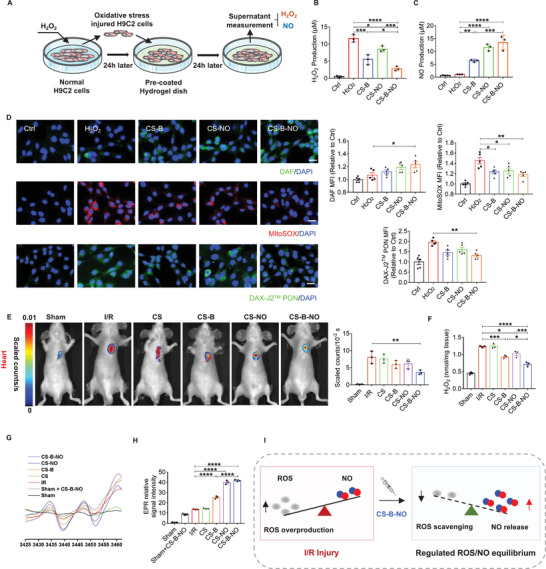
CS‐B‐NO hydrogel modulated the myocardial ROS/NO disequilibrium after I/R injury by simultaneously releasing NO and scavenging ROS. A) Schematic illustration of H9C2 cells oxidative stress model of H_2_O_2_‐induced oxidative. B,C) Quantitative analysis of H_2_O_2_ and NO release with different treatments one day after oxidative stress stimuli (n = 3 individual experiments). D) After 24 h of H_2_O_2_ stimulation followed with different hydrogel treatments, H9C2 cells were stained with DAF‐FM DA, MitoSOX Red, and DAX‐J2 PON Green 99 to detect the production of NO, superoxide, and peroxynitrite (ONOO^−^), respectively. The mean fluorescence intensity was quantified respectively. (scale bar = 20 µm, n = 5 individual experiments). E) In vivo imaging of H_2_O_2_ and quantitative analysis of fluorescence intensity. F) Quantitative analysis on the H_2_O_2_ level of heart homogenate after different treatments at one‐day post‐surgery (n = 3). G) Representative EPR spectra reflecting NO generation in the presence of (DETC)_2_Fe. H) NO levels were determined by quantitation of (DETC)_2_Fe‐NO complex using 2,2,5,5‐tetramethyl piperidine 1‐oxyl (TEMPO), n = 3 animals for each group. I) Schematic illustration demonstrating that CS‐B‐NO hydrogel treatment restored the local balance of ROS/NO in infarcted myocardium after I/R injury. Data are expressed as mean ± SEM. Significant differences were detected by one‐way ANOVA with Tukey's multiple comparisons test, *p<0.05, **p<0.01, ***p<0.001, ****p<0.0001.

To determine the regulatory effect of the CS‐B‐NO hydrogel on ROS/NO disequilibrium after I/R injury, an in vitro oxidative stress model was established by treating H9C2 cells with H_2_O_2_ for 24 h and then subjecting the cells to different treatments (**Figure** **2**A). The levels of H_2_O_2_ and NO in the cell culture supernatants were monitored with an H_2_O_2_ assay kit and chemiluminescence NO analyzer (NOA), respectively. The results showed that oxidative‐stress induced the overproduction of ROS (H_2_O_2_), resulting in the disequilibrium of ROS and NO (ROS>NO). Administration of the CS‐B and CS‐NO hydrogels was proven to be an effective strategy to correct the ROS/NO balance through direct clearance of H_2_O_2_ or production of NO, respectively. A more prominent effect was observed in the group treated with the CS‐B‐NO hydrogel, which could simultaneously scavenge H_2_O_2_ and release NO. As a result, the CS‐B‐NO hydrogel significantly (*p*<0.0001) reduced the H_2_O_2_ level but enhanced the NO level compared to those in the group without treatment, reversing the balance between ROS and NO (ROS<NO) to resemble the balance characteristic of a normal physiological state (Figure 2B,C). It is worth noting that the level of NO has been remarkably enhanced after CS‐B treatment. This is because CS‐B treatment provides direct clearance of H_2_O_2_ that reduces the tendency of ONOO^−^ formation, therefore it is reasonable to ascribe the enhanced NO level in CS‐B group to the increased bioavailability of endogenous NO generated by cardiomyocytes, H9C2 cells.^[^ ^19^ ^]^

CS‐NO is a NO‐releasing biomaterial reported by our group before,^[^ ^6^ ^]^ in which galactose‐protected NONOate moieties were grafted onto the natural chitosan backbone. As a result, CS‐NO demonstrates a high degree of stability in PBS without detectable NO production and is decomposed to release NO in the presence of glycosidase secreted by the cells (Figure S10, Supporting Information).

The production of superoxide (O_2_^•−^) and NO in H9C2 cells was further evaluated by fluorescence staining with MitoSOX Red and DAF‐FM DA, respectively. As shown in Figure 2D, faint red and green fluorescence was observed in H9C2 cells under normal culture conditions. Oxidative stress injury led to red fluorescence due to the production of superoxide (O_2_^•−^), and the fluorescence density gradually decreased in sequence when CS‐B, CS‐NO, and CS‐B‐NO were applied. In contrast, green fluorescence, indicating NO, followed the opposite trend, with the highest density observed upon treatment with CS‐B‐NO. In addition, NO is scavenged by O_2_^•−^ leading to the formation of the powerful oxidant, peroxynitrite (ONOO^−^). The production of ONOO^−^ was also detected by the fluorescent probe (DAX‐J2 PON Green 99). CS‐B‐NO significantly inhibited the formation of ONOO^−^ because of the simultaneous ROS scavenging and NO release.

### 2.3 The CS‐B‐NO Hydrogel Modulated Myocardial ROS/NO Disequilibrium After I/R Injury

We then investigated the regulatory effect of CS‐B‐NO on the dysregulated ROS/NO balance in a mouse model of myocardial I/R injury. H_2_O_2_ generation in the infarcted heart was evaluated by live imaging using an H_2_O_2_‐sensitive near‐infrared fluorescent probe (C_46_H_47_BBrNO_6_) (Figure 2E; Figure S3, Supporting Information). Compared to that in the sham group, the fluorescent signal was markedly elevated after I/R injury, and treatment with the CS‐B‐NO hydrogel significantly (*p*<0.01) lowered the H_2_O_2_ level. Notably, the fluorescence density in the CS‐B and CS‐NO groups was also moderately decreased due to the antioxidant function via ROS scavenging or NO release by the hydrogel. Moreover, the delivery of NO by the hydrogel quenched endogenous H_2_O_2_. Next, the level of H_2_O_2_ in heart tissues was quantified with an H_2_O_2_ assay kit (Figure 2F), and the lowest H_2_O_2_ production was observed in the CS‐B‐NO group because of the dual effects of CS‐B‐NO, which eliminated ROS and generated NO.

Additionally, regional NO generation in the heart after I/R was evaluated by electron paramagnetic resonance (EPR) using ferrous *N*‐diethyl dithiocarbamate (Fe‐DETC) as the spin‐trapping reagent. The resultant NO adduct, (DETC)_2_Fe‐NO, exhibited a characteristic triplet EPR signal (a_N_ = 13.06 G, g_iso_ = 2.041) at room temperature (Figure 2G). Quantitative analysis indicated that NO levels were moderately enhanced in both the group subjected to I/R and the group subjected to I/R and treated with the plain CS hydrogel (without therapeutic function) compared to the sham group due to endogenous NO production after I/R injury. A greater increase was observed in the CS‐B‐NO and CS‐NO groups, in which NO levels were significantly (*p*<0.0001) higher than those in the I/R group. In addition, the generation of NO from CS‐B‐NO was approximately three times higher in the I/R group than in the sham group, indicating that the NO‐release behavior was controlled by the local H_2_O_2_ level (Figure 2H). H_2_O_2_ attacks the boronic ester group directly and transforms to boric acid pinacol ester, then a self‐immolative cascade reaction occurs, leading to the release of NO (Figure 1A). Hence, the amount of NO released from CS‐B‐NO is proportional to the level of ROS. As a result, it enables the release responsiveness of CS‐B‐NO to the pathologic microenvironment—a mechanism that restricts the undesirable overproduction of NO. In contrast, *β*‐glycosidase secreted by the cells acts as a catalyst for the hydrolysis of the glucosidic bond, enabling the further release of NO from CS‐NO. Accordingly, the level of endogenous *β*‐glycosidase cannot affect the releasing amount of NO from CS‐NO although it also shows an increasing tendency after I/R injury.^[^ ^20^ ^]^ Our results further reflected that the amount of NO released from CS‐NO did not show significant variation between sham and I/R groups (Figure S11, Supporting Information).

NO overdosage was reported to cause serious adverse events, but the widely accepted range for the physiological concentration of NO is 10 nm to 1 µm.^[^ ^21^ ^]^ In this study, after comparisons of EPR results (Figure 2G) against a standard curve, it was shown that NO production in vivo falls within the nanomolar concentration range that is favorable to cardioprotection.

Collectively, these results support the hypothesis that administration of the CS‐B‐NO hydrogel efficiently modulated local concentrations of ROS and NO after I/R injury by simultaneously releasing NO and scavenging H_2_O_2_ (Figure 2I).

### 2.4 The CS‐B‐NO Hydrogel Ameliorated Myocardial I/R Injury in Mice at an Early Stage

**Figure 3:**
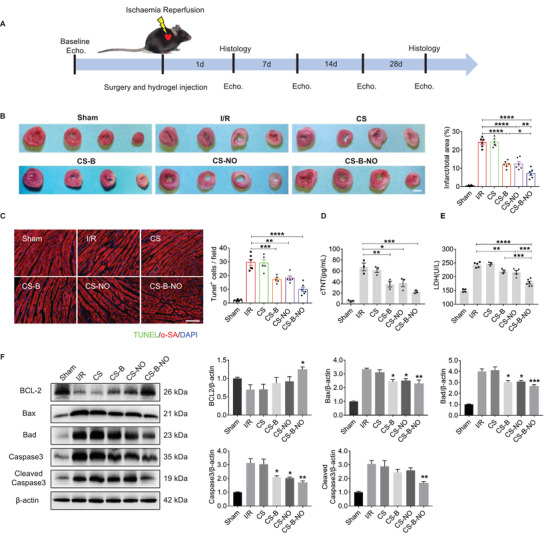
CS‐B‐NO hydrogel ameliorated myocardial I/R injury in mice at early stage. A) Experimental schedule for the treatment of I/R injury via intramyocardial injection of CS‐B‐NO hydrogel. B) TTC staining (scale bar = 2 mm), was performed to evaluate the ischemia injured myocardium via quantifying the infarct area (n = 6 animals per group). C) Representative images of TUNEL staining at 1 day after surgery to detect apototic nucleus (scale bar = 100 µm), and positive staining nucleus was quantified (n = 5–6 animals per group). D,E) Serum cTnT, LDH levels were analyzed, respectively (n = 3–6 animals per group). F) Representative Western blot images and quantitative data showing the expression of BCL2, Bax, Bad, Caspase3, cleaved Caspase3 in the hearts at 3 days after different treatments (n = 5 animals per group). Data are expressed as mean ± SEM. Significant differences were detected by one‐way ANOVA with Tukey's multiple comparisons test, *p<0.05, **p<0.01, ***p<0.001, and ****p<0.0001.

TTC staining was utilized to compare the extent of cardiac damage 2 days after the initiation of reperfusion among the different groups. The results showed that injection of CS‐B and the CS‐NO hydrogel moderately reduced the damaged area, and the infarct area was shown to be significantly (*p*<0.05, 0.01, or 0.0001) decreased in the mice treated with the CS‐B‐NO hydrogel compared with the other groups (**Figure** **3**B). Then, we performed terminal deoxynucleotidyl transferase dUTP nick‐end labeling (TUNEL) staining after 1 day to investigate I/R‐induced cardiomyocyte apoptosis. As shown in Figure 3C, I/R stress led to an increase in cardiomyocyte apoptosis compared with that in the sham group. After treatment with the CS‐B and CS‐NO hydrogels, the number of TUNEL‐positive cardiomyocytes was remarkably decreased (CS‐B: *p*<0.001 vs the I/R group; CS‐NO: *p*<0.01 vs the I/R group). Moreover, fewer TUNEL^+^ cardiomyocytes were observed after CS‐B‐NO hydrogel injection. The desired anti‐apoptotic effect of CS‐B‐NO was also confirmed by flow cytometric assessment of the cardiomyocytes of neonatal mice stimulated with H_2_O_2_ in vitro (Figure S12, Supporting Information).

Both serum cardiac troponin T (cTnT) and lactate dehydrogenase (LDH) are widely utilized as potential biomarkers to evaluate the ischemic severity of the myocardial injury.^[^ ^22^ ^]^ Compared with those in the sham group, I/R injury induced a marked elevation in serum cTnT and LDH levels. Treatment with the CS‐B and CS‐NO hydrogels reduced the levels of serum cTnT and LDH, while the lowest cTnT and LDH levels were observed in I/R mice injected with the CS‐B‐NO hydrogel, which further confirmed the protective effect of the CS‐B‐NO hydrogel against cardiac injury (Figure 3D,E). Furthermore, the expression of apoptosis‐related genes was analysed by Western blot analysis and RT‐PCR. The expression of the anti‐apoptotic molecule BCL‐2 was upregulated, whereas that of the pro‐apoptotic molecules Bax, Bad, Caspase 3, and cleaved Caspase3 was downregulated at both the gene and protein levels after CS‐B‐NO treatment (Figure 3F; Figure S13, Supporting Information).

### 2.5 The CS‐B‐NO Hydrogel Improved Heart Function and Promoted Heart Repair at 28 Days

**Figure 4:**
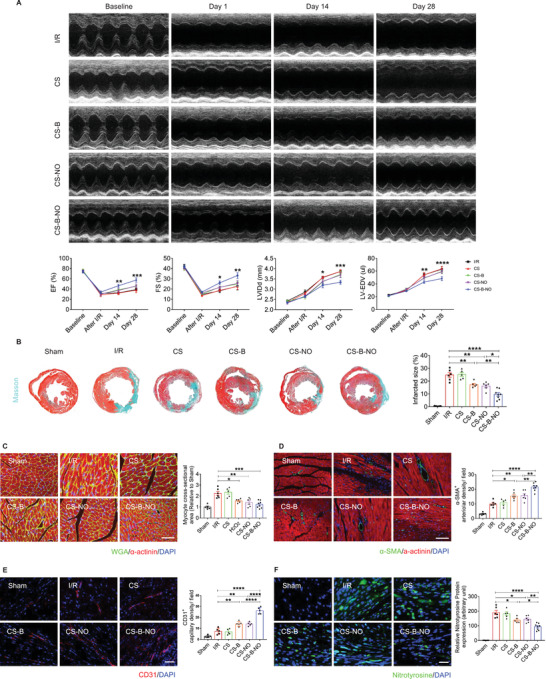
CS‐B‐NO hydrogel improved heart function, stimulated angiogenesis, and reduced adverse cardiac remodeling in mice after myocardial I/R injury. A) Cardiac echo measurement was performed at different time‐points post‐surgery, and cardiac function indicators of left ventricular‐ejection fraction (LV‐EF), left ventricular‐fractional shortening (LV‐FS), left ventricular internal diameter at end diastole (LVIDd), and left ventricular end‐diastolic volume (LV‐EDV) were evaluated accordingly. Data are expressed as mean ± SEM, n = 5–8 animals per group. Significant differences were detected by two‐way ANOVA with Tukey's multiple comparisons test, *p<0.05, **p<0.01, ***p<0.001, and ****p<0.0001. The hearts were collected for histological analyses at 28 days post‐surgery. B) Masson's trichrome (scale bar = 400 µm) was performed, and infarcted size was quantified accordingly. C) Representative images of WGA immunofluorescence staining and the cross‐section area of cardiomyocytes were measured (scale bar = 50 µm). D) Representative images of *α*‐SMA immunofluorescence staining and number of the *α*‐SMA positive arterioles were counted (scale bar = 100 µm). E) Representative images of CD31 immunofluorescence staining and number of the CD31 positive capillaries were quantified (scale bar = 50 µm). F) Representative images of nitrotyrosine immunostaining and quantification of the relative nitrotyrosine protein expression (scale bar = 25 µm). Data are expressed as mean ± SEM, n = 5–8 animals for each group. Significant differences were detected by one‐way ANOVA with Tukey's multiple comparisons test, *p<0.05, **p<0.01, ***p<0.001, and ****p<0.0001.

The impact of the hydrogel treatments on cardiac function after I/R surgery was measured by echocardiography at the indicated time points. Injection of the CS‐B‐NO hydrogel improved cardiac ejection function, as shown by increases in the left ventricular ejection fraction (LV‐EF) and LV fractional shortening (LV‐FS). Moreover, cardiac dimensions were also well maintained. Four weeks after I/R injury, the dilated internal diameter (LVIDd) and the end‐diastolic volume (LV‐EDV) of the left ventricle were increased in the I/R mice, indicating dilated cardiomyopathy. However, treatment with the CS‐B‐NO hydrogel significantly suppressed dilation of the heart (LVIDd: *p*<0.001 vs the I/R group; LV‐EDV: *p*<0.0001 vs the I/R group) (**Figure** **4**A).

Next, histological analyses were performed to investigate the long‐term impact of CS‐B‐NO hydrogel treatment on cardiac remodeling after I/R injury. Consistent with the improvement in cardiac function, cardiac morphology was also improved by the CS‐B‐NO hydrogel. Masson's trichrome staining showed an approximately three times smaller infarct size in the CS‐B‐NO group than in the I/R and CS groups (Figure 4B). In addition, histological analyses of heart tissue sections showed obvious fibrillary layers in the I/R and CS‐treated groups. In contrast, moderate thickening of the muscle was observed in the CS‐B and CS‐NO groups, and the infarcted regions in the CS‐B‐NO‐treated mice retained distinct and thick muscle layers (Figure 4B; Figure S14, Supporting Information). Moreover, extensive collagen deposition was observed in both the I/R and CS hydrogel‐treated mice, but this was clearly inhibited after CS‐B‐NO treatment (Figure S15, Supporting Information).

The occurrence of cardiomyocyte hypertrophy was evaluated by using WGA staining. In general, cardiomyocyte hypertrophy was significantly increased, while treatment with the functional hydrogels (CS‐NO, CS‐B, and CS‐B‐NO) effectively reduced the cross‐sectional area of cardiomyocytes in I/R hearts, with the most pronounced inhibitory effect observed upon treatment with the CS‐B‐NO hydrogel (Figure 4C).

We then assessed the pro‐angiogenic effect of the CS‐B‐NO hydrogel in the heart after ischemic injury. Treatment with the functional hydrogels (CS‐B, CS‐NO, and CS‐B‐NO) effectively promoted neovascularization at four weeks after I/R injury compared to that in the I/R and CS groups. More importantly, the numbers of both *α*‐SMA^+^ arterioles and CD31^+^ capillaries distributed at the infarcted border zone were significantly (*p*<0.01, *p*<0.0001) enhanced in the hearts of the CS‐B‐NO group compared with those of the groups treated with the CS‐NO and CS‐B hydrogels. (Figure 4D,E). In addition, the expression of eNOS after different hydrogel treatments was assessed, and the CS‐B‐NO hydrogel effectively promoted eNOS expression after IR injury (Figure S16, Supporting Information).

During I/R‐induced myocardial injury, disruption of the balance between O_2_^•−^ and NO in mitochondria often leads to the formation of the highly reactive oxidant ONOO^−^, which can mediate cardiac dysfunction and cell death in various types of reperfusion injury.^[^ ^23^ ^]^ Due to the short half‐life of ONOO^−^, measurement of the more stable ONOO^−^‐mediated nitration product nitrotyrosine has become an acceptable and reliable biomarker for the detection of ONOO^−^ in vivo. As shown in Figure 4F, in normal heart tissue, little if any positive staining was observed. In contrast, strong positive green staining for nitrotyrosine was observed within the reperfused myocardium in both the I/R and CS groups, whereas CS‐B and CS‐NO hydrogel treatment greatly suppressed the expression of nitrotyrosine compared to that in the I/R group (CS‐B: *p*<0.05, CS‐NO: *p*<0.05). More significant (*p*<0.0001) inhibition was observed in the CS‐B‐NO group (Figure 4F).

### 2.6 The CS‐B‐NO Hydrogel Modulated the Cardiac Inflammatory Response by Inhibiting the NF‐*κ*B Signaling Pathway

**Figure 5:**
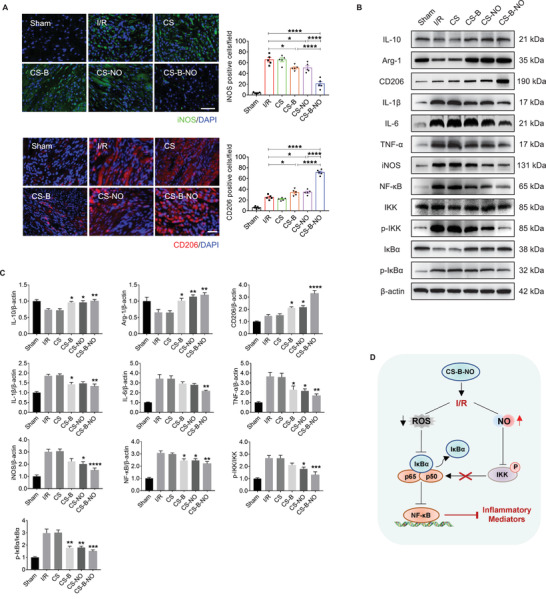
CS‐B‐NO hydrogel inhibited NF‐*κ*B inflammatory signaling pathway by regulating local ROS/NO balance after myocardial I/R injury. A) Macrophage polarization was detected by immunofluorescence staining targeting iNOS and CD206, the markers of M1 and M2 macrophage phenotype, respectively (scale bar = 50 µm). B,C) Representative Western blot images and quantitative data showing the expression of IL‐10, Arg‐1, CD206, IL‐1*β*, IL‐6, TNF‐*α*, iNOS, NF‐*κ*B, IKK, p‐IKK, I*κ*B*α*, and p‐I*κ*B*α*, in the hearts after I/R injury with different treatments. Data are expressed as mean ± SEM, n = 5 animals for each group, Significant differences were detected by one‐way ANOVA with Tukey's multiple comparisons tests, *p<0.05, **p<0.01, ***p<0.001, and ****p<0.0001. D) Schematic illustration summarizing the mechanism of CS‐B‐NO hydrogel on inhibiting the NF‐*κ*B signaling pathway after I/R injury.

Macrophages are the major players in inflammation resolution and reparative transition. The modulatory effect of the CS‐B‐NO hydrogel on macrophage polarization was first evaluated by immunofluorescence staining with iNOS and CD206, markers of pro‐inflammatory M1 macrophages, and reparative M2 macrophages, respectively. Treatment with the functional hydrogels (CS‐B, CS‐NO, and CS‐B‐NO) effectively increased the infiltration of M2 macrophages into the heart while inhibiting that of M1 macrophages; a 2.84‐fold increase and a 3.14‐fold reduction, respectively, were observed in the CS‐B‐NO group (**Figure** **5**A). Western blotting was also performed to detect the expression of inflammatory‐related mediators in the heart after I/R injury. The CS‐B‐NO hydrogel demonstrated the most efficient immunoregulatory effect; that is, the levels of pro‐inflammatory cytokines secreted by M1 macrophages (IL‐1*β*, IL‐6, TNF‐*α*, and iNOS) were significantly (*p*<0.01 or 0.0001) decreased, whereas the levels of M2‐related anti‐inflammatory cytokines (IL‐10, Arg‐1, and CD206) were significantly (*p*<0.01 or 0.0001) enhanced after CS‐B‐NO hydrogel treatment (Figure 5B,C). The results were further supported by the results of an RT‐PCR assay (Figure S17, Supporting Information).

Myocardial I/R leads to a burst of oxidative stress that produces an excess of ROS, augmenting inflammation. The NF‐*κ*B signaling pathway plays a major role in innate immunity and inflammatory responses. Western blot analyses revealed that I/R injury induced NF‐*κ*B activation and enhanced the phosphorylation of IKK and I*κ*B*α*. After treatment with the functional hydrogels (CS‐B, CS‐NO, and CS‐B‐NO), the expression of NF‐*κ*B was remarkably downregulated (CS‐B: *p*<0.05, CS‐NO: *p*<0.05, CS‐B‐NO: *p*<0.01 vs the I/R group). More specifically, CS‐NO treatment decreased NF‐*κ*B activation mainly by inhibiting the phosphorylation of IKK and subsequent H_2_O_2_‐induced I*κ*B*α* phosphorylation during I/R injury (*p*<0.05 or 0.01).^[^ ^24^ ^]^ The inhibitory effect in the CS‐B hydrogel‐treated group was achieved by directly reducing I*κ*B*α* phosphorylation by H_2_O_2_ elimination (*p*<0.01) (Figure 5B,C). In contrast, the CS‐B‐NO hydrogel had the most effective inhibitory effect on NF‐*κ*B activation by simultaneously decreasing the phosphorylation of both IKK and I*κ*B*α*. Cellular experiments using H_2_O_2_‐stimulated H9C2 cells also supported these in vivo observations (Figure S18, Supporting Information). Generally, these results suggest that the CS‐B‐NO hydrogel protected against inflammation‐induced heart injury, most likely by inhibiting activation of the NF‐*κ*B signaling pathway and downstream macrophage polarization and inflammatory factor expression (Figure 5D).

### 2.7 The CS‐B‐NO Hydrogel Protected Against Oxidative Stress Induction After I/R Injury Via Adaptive Regulation of the Nrf2‐Keap1 Signaling Pathway

**Figure 6:**
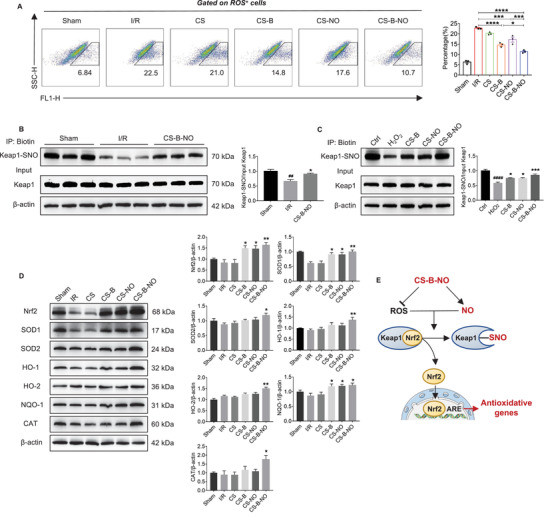
CS‐B‐NO hydrogel ameliorated oxidative stress induced by ischemic/reperfusion injury via adaptive regulation of the Nrf2‐Keap1 signaling pathway. A) Flow cytometric analysis was performed to quantify the number of ROS positive cells in myocardial tissue at 1‐day post‐surgery (n = 3 animals per group). B) Mice after I/R injury were treated by myocardial injection of CS‐B‐NO hydrogel, and 7 days post‐surgery Keap1 S‐nitrosylation of the heart tissues was detected by biotin switch (^##^ *p*<0.01 vs Sham, *p<0.05 vs I/R, n = 5 animals per group). C) H9C2 cells were stimulated with H_2_O_2_ for 24 h and followed by various treatments. Keap1 S‐nitrosylation was further detected by biotin switch assay. (^####^ *p* <0.0001 vs Ctrl, *p<0.05, ***p<0.001 vs H_2_O_2_ stimulated group, n = 5 individual experiments) D) Representative Western blot images and quantitative data showing the expression of Nrf2, SOD1, SOD2, HO‐1, HO‐2, NQO‐1, and CAT in mice hearts in mice hearts after I/R injury with different treatments (n = 5 animals per group). Data are expressed as mean ± SEM, Significant differences were detected by one‐way ANOVA with Tukey's multiple comparisons test, *p<0.05, **p<0.01, ***p<0.001, and ****p<0.0001. E) Schematic illustration summarizing the mechanism of CS‐B‐NO hydrogel on activation of the Nrf2 pathway against oxidative stress via enhancing Keap1 S‐nitrosylation.

Nrf2 is an important cytoprotective transcription factor. Many findings suggest that Nrf2 plays a vital role in defense against oxidative stress by activating cellular antioxidant mechanisms and inhibiting pro‐inflammatory signaling. In this study, we first assessed the protective effect of CS‐B‐NO against oxidative stress. Flow cytometric assays demonstrated that the functional hydrogels clearly decreased the proportion of ROS‐positive cells in heart tissues after I/R injury, with the most significant (*p*<0.0001) reduction observed in the CS‐B‐NO group (**Figure** **6**A). The ROS^+^ cells in myocardial tissue at 1 day post‐surgery were further identified by co‐immunostaining with markers of cardiomyocytes (cTNI), cardiac fibroblasts (Vimentin), and M1 macrophages (iNOS), respectively, which were the main cell types of myocardial tissue. The results showed that cardiac fibroblasts and macrophages are the major contributors to ROS production after I/R injury, while the CS‐B‐NO hydrogel treatment significantly reduced the percentage of ROS positive cells in the myocardium (*p*<0.0001) (Figure S19, Supporting Information). Oxidative stress was also found to be mitigated in the cardiomyocytes of neonatal mice stimulated with H_2_O_2_ in vitro and then treated with the functional hydrogels, especially CS‐B‐NO (Figure S20, Supporting Information).

Mechanistically, NO has been reported to activate Nrf2 by S‐nitrosylation of Keap1.^[^ ^25^ ^]^ Therefore, we purified the S‐nitrosylated proteins from the hearts of I/R mice after various treatments through biotin switching. Keap1 has been identified as a highly S‐nitrosylated protein, and after I/R injury, Keap1 S‐nitrosylation was clearly decreased (*p*<0.01 vs the sham group). However, treatment with the CS‐B‐NO hydrogel efficiently increased Keap1 S‐nitrosylation compared to that of the I/R group (*p*<0.05 vs the I/R group)(Figure 6B). A similar phenomenon was observed in in vitro experiments using H_2_O_2_‐stimulated H9C2 cells (Figure 6C). More importantly, the increase in Keap1 S‐nitrosylation was more pronounced in the CS‐B‐NO group than in the other two groups (CS‐B and CS‐NO). To further elucidate the underlying mechanism, we determined the free thiol content in heart tissues by monobromobimane fluorescence assay.^[^ ^26^ ^]^ As shown in Figure S21, Supporting Information, I/R injury clearly reduced the quantity of free thiols, thus restricting further S‐nitrosylation of the corresponding proteins. As a result, the effect of NO was diminished in the CS‐NO group. In contrast, in addition to its ability to deliver NO, CS‐B‐NO could simultaneously scavenge local H_2_O_2_. This decrease in the ROS level further reduced the oxidative post‐translational modification of free thiols in cysteines, making them available for S‐nitrosylation.^[^ ^27^ ^]^ The enhanced Keap1 S‐nitrosylation in the CS‐B group could also be explained by the increased bioactivity of endogenous NO following H_2_O_2_ scavenging.

Next, we detected the expression of Nrf2 and its downstream target genes. As expected, Nrf2 was clearly increased in the I/R mice treated with the CS‐B and CS‐NO hydrogels (CS‐B: *p*<0.05, CS‐NO: *p*<0.05 vs the I/R group), especially in the group treated with the CS‐B‐NO hydrogel (*p*<0.01). Accordingly, the expression of the Nrf2‐related antioxidant defense enzymes, including superoxide dismutase (SOD), haem oxygenase (HO), quinone oxidoreductase 1 (NQO1), and catalase, was significantly (*p*<0.05 or 0.01) upregulated in the CS‐B‐NO group (Figure 6D). In vitro cell assays also demonstrated the expression of Nrf2 and downstream antioxidant‐related proteins was enhanced at both the gene and protein levels after functional hydrogel treatment (Figures S22 and S23, Supporting Information).

Collectively, all these results indicate that administration of a functional hydrogel with dual functions in NO delivery and H_2_O_2_ scavenging, that is, CS‐B‐NO, is an effective strategy to activate the Nrf2 signaling pathway via the enhanced S‐nitrosylation of Keap1.

### 2.8 Nrf2 is Required for CS‐B‐NO Hydrogel‐Mediated Myocardial Protection During Myocardial I/R

**Figure 7:**
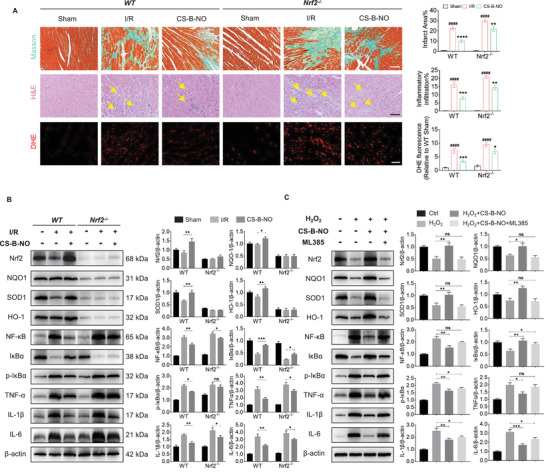
Nrf2 deficiency abrogates the cardioprotection of CS‐B‐NO hydrogel in mice after ischemic/reperfusion injury. A) H&E (scale bar = 100 µm) and DHE staining (scale bar = 50 µm) were performed in wild‐type C57BL/6 mice (WT) and Nrf2 deficient mice (Nrf2^−/−^) at 3 days post‐surgery. Myocardium fibrosis was indicated by yellow arrows. Masson's trichrome was performed at 28 days post‐surgery (scale bar = 100 µm). The infarction area, inflammatory cell infiltration, and DHE positive staining were quantified, respectively. (^####^ *p*<0.0001 vs Sham, *p<0.05, **p<0.01, ***p<0.001, ****p<0.0001 vs I/R, n = 5 animals per group). B) WT and Nrf2^−/−^ mice after I/R surgery were treated by CS‐B‐NO hydrogel. At 7 days post‐surgery, western blotting analysis was performed to quantify the expression level of Nrf2, NQO1, SOD1, HO‐1, NF‐*κ*B, I*κ*B*α*, p‐I*κ*B*α*, TNF*α*, IL‐1*β*, and IL‐6 in hearts (n = 5 animals per group). C) H9C2 cells were treated with 5 µm ML385 to inhibit Nrf2 expression in vitro. 200 µm H_2_O_2_ was introduced to stimulate H9C2 cells with or without Nrf2 inhibition for 24 h, followed by administration of CS‐B‐NO hydrogel for 48 h. Protein expression level of Nrf2, NQO1, SOD1, HO‐1, NF‐*κ*B, I*κ*B*α*, p‐I*κ*B*α*, TNF*α*, IL‐1*β*, and IL‐6 in H9C2 cells was evaluated by Western blotting analysis (n = 5 individual experiments). Data are expressed as mean ± SEM. Significant differences were detected by one‐way ANOVA with Tukey's multiple comparisons test, *p<0.05, **p<0.01, ***p<0.001, and ****p<0.0001.

To gain further insight into the pathophysiological importance of Nrf2 activation by NO‐induced Keap1 S‐nitrosylation, we detected the regulatory effect of the CS‐B‐NO hydrogel on oxidation and inflammation in Nrf2^−/−^ mice. Histological analyses (Masson's trichrome, HE, and DHE staining) revealed that Nrf2 deficiency aggravated myocardium fibrosis, the inflammatory response, and oxidative stress in the I/R mice (**Figure** **7**A). Similarly, the induction of NQO1, SOD1, and HO‐1 by the CS‐B‐NO hydrogel was totally abolished in the Nrf2^−/−^ mice (Figure 7B). The inhibitory effect of the CS‐B‐NO hydrogel on the NF‐*κ*B signaling pathway was also less pronounced in the Nrf2^−/−^ mice than in their wild‐type counterparts. As a result, expression of inflammatory cytokines, such as IL‐1*β*, IL‐6, and TNF*α*, was induced to a greater degree in the Nrf2^−/−^ mice than in the WT mice.

In vitro experiments were further performed by using an Nrf2 inhibitor (ML385) to inhibit Nrf2 in H9C2 cells. The expression of antioxidant genes related to Nrf2 signaling and inflammatory genes corresponding to NF‐*κ*B signaling was also detected (Figure 7C). The results demonstrated that the inhibition of Nrf2 diminished the antioxidant function and resulting cardioprotection provided by the CS‐B‐NO hydrogel. Moreover, Nrf2 deficiency exacerbated the NF‐*κ*B‐mediated pro‐inflammatory response.

## 3 Discussion

NO is a key signaling molecule in the cardiovascular, immune, and central nervous systems. NO produced in endothelial cells and cardiomyocytes is important in regulating cardiac function. The heart expresses all three NO synthase (NOS) isoforms, and NOS3 (endothelial NOS) is located in the endothelium and cardiomyocyte caveolae, where it demonstrates anti‐inflammatory, vasoactive, and vasoprotective effects.^[^ ^28^ ^]^ In addition to the vasoactive functions of NO, increasing attention has been given to the protective role of NO against cardiac ischemia, the underlying mechanism of which has been suggested to be mitochondria‐targeted cardioprotection,^[^ ^29^ ^]^ including the opening of ATP‐sensitive potassium channels and inhibition of mitochondrial respiration and electron transfer.^[^ ^8^, ^15^ ^]^

**Figure 8:**
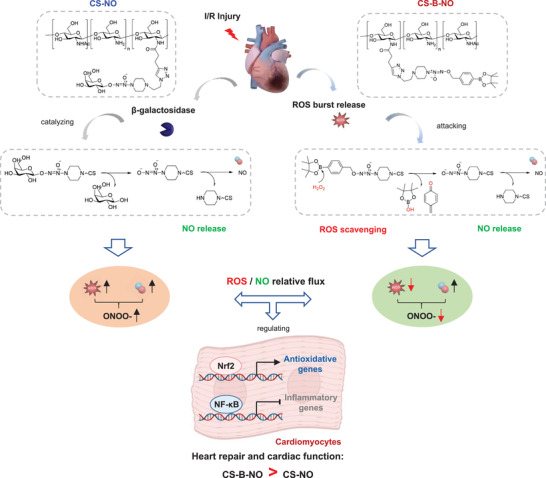
Mechanistic summary of CS‐B‐NO hydrogel for myocardial repair after ischemic/reperfusion injury by a head‐to‐head comparison with CS‐NO.

In a previous study, we prepared a NO‐releasing biomaterial (CS‐NO) that releases NO under the catalysis of *β*‐glycosidase.^[^ ^6^ ^]^ Enzyme‐controlled release of NO efficiently promotes vascularization, thus showing great promise in the therapy of various ischemia diseases, including MI.^[^ ^7b^ ^]^ In the present study, we designed and synthesized a chitosan‐based hydrogel that releases NO under the attack of H_2_O_2_. Hence it holds both ROS‐scavenging and NO‐delivery dual functions. The therapeutic efficacy of it was systemically evaluated in a mouse model of myocardial I/R injury that enables a head‐to‐head comparison with CS‐NO. Administration of CS‐B‐NO effectively delivered NO to the infarcted myocardium while lowering the level of ROS as well as peroxynitrite (ONOO^−^), a potent oxidant, in contrast to CS‐NO which only provided NO delivery function. As a result, the local ROS/NO balance was effectively regulated, leading to enhanced therapeutic efficacy in CS‐B‐NO (**Figure** **8**). Although only supplementation with exogenous NO also inhibited ROS‐dependent oxidation depending on relative ROS flux,^[^ ^30^ ^]^ therapeutic efficacy of NO was markedly compromised in CS‐NO.

Mechanistically, we also found that the ROS/NO equilibrium plays an essential role in regulating oxidative stress and inflammation,^[^ ^31^ ^]^ which are considered the most important molecular mechanisms responsible for I/R injury.

First, thiols are critical sites at which NO and O_2_^•−^ interact in biological systems; hence, the relative flux of ROS and NO can determine the post‐translational modification of thiol‐containing proteins through nitrosation and oxidation, which further influence cellular function.^[^ ^32^ ^]^ Protein S‐nitrosylation, binding of an NO moiety with the thiol group of a cysteine residue to form an S‐nitrosothiol (SNO) group, has been shown to be a critical regulator of cardiovascular function and an important contributor to NO‐mediated cardioprotection.^[^ ^33^ ^]^ The S‐nitrosylation of caspases by NO can inhibit apoptosis, which plays an important role in regulating the function of mitochondrial caspase.^[^ ^34^ ^]^ Previous studies have demonstrated that post‐translational S‐nitrosylation inhibits mitochondrial complex I, reducing ROS generation during reperfusion.^[^ ^35^ ^]^ The presence of NO in ischemic preconditioning (IPC) results in protein S‐nitrosylation, which is involved in the regulation of mitochondrial energetics and calcium transport.^[^ ^36^ ^]^ Lin et al. suggested that long‐term estrogen exposure protects hearts largely via the activation of ER‐beta and NO/SNO signaling.^[^ ^37^ ^]^

The Keap1‐nuclear factor‐erythroid 2‐related factor 2 (Nrf2) pathway is a redox‐sensitive signaling pathway that facilitates transcriptional regulation of a family of modulatory and cytoprotective redox genes that facilitate the defense against oxidative stress.^[^ ^38^ ^]^ However, a high ROS concentration causes mitochondrial dysfunction, thus suppressing Nrf2‐Keap1 pathway activation, which in turn leads to cellular necrosis, apoptosis, and further cardiac injury. Herein, we have shown that the CS‐B‐NO hydrogel efficiently mediated Keap1 S‐nitrosylation by regulating local ROS/NO disequilibrium after I/R injury, which led to the release of Nrf2 from a complex with Keap1. Upon release, Nrf2 was found to be phosphorylated and transported to the nucleus, where it bound a promoter with the ARE sequence. Accordingly, it activated target genes that encode proteins that scavenge ROS and exerted antagonistic effects on the NF‐*κ*B signaling pathway, which plays a dominant role in protecting the heart against oxidative stress after I/R injury. However, the specific sites of Keap1 that are targeted for S‐nitrosylation by NO remain to be further identified.

Inflammation is an important factor involved in the pathological evolution of heart disease. NF‐*κ*B, a key transcription factor, is known to regulate the expression of many genes involved in the immune response, inflammation, viral infection, and programmed cell death.^[^ ^39^ ^]^ Phosphorylation by inhibitor of nuclear factor NF‐*κ*B (I*κ*B) kinase (IKK) promotes the ubiquitylation and proteasomal targeting of I*κ*B, which allows NF‐*κ*B to translocate to the nucleus and initiate transcription. S‐Nitrosylation of a critical cysteine residue within IKK*β* inhibits I*κ*B phosphorylation; in addition, S‐nitrosylation of NF‐*κ*B with exogenous NO or NO produced due to iNOS induction inhibits NF‐*κ*B‐dependent DNA binding, promoter activity, and gene transcription.^[^ ^40^ ^]^ Our results demonstrated the inhibitory effects of the CS‐B‐NO hydrogel on the NF‐*κ*B signaling pathway. The CS‐B‐NO hydrogel inhibited the activation of NF‐*κ*B signaling and downstream pro‐inflammatory factors by downregulating the phosphorylation of IKK/I*κ*B*α*, which further promoted the polarization of macrophages into the M2 phenotype. NF‐*κ*B also contributes to the hypertrophic growth of cardiomyocytes and resultant heart failure.^[^ ^41^ ^]^ Our findings also suggest that CS‐B‐NO hydrogel treatment efficiently limited the occurrence of cardiomyocyte hypertrophy and myocardial fibrosis after I/R injury, which could be attributed to the inhibition of NF‐*κ*B signaling.

In addition, Nrf2 and NF‐*κ*B signaling regulate physiological cellular redox homeostasis and the responses to stress and inflammation. Growing evidence supports molecular cross‐talk between these two important pathways to maintain redox equilibrium. As levels of the Nrf2 protein increase, the transcription of Nrf2 limits NF‐*κ*B activity in part by providing a reductive microenvironment.^[^ ^42^ ^]^ Pharmacological and genetic studies have shown the presence of functional cross‐talk between these two important pathways. In a variety of experimental models, studies have shown that Nrf2 plays a key role in inhibiting the NF‐*κ*B‐driven inflammatory response.^[^ ^43^ ^]^ At the transcriptional level, NF‐*κ*B represses the expression of Nrf2 target genes through competition with Nrf2 for the transcriptional co‐activator CREB‐binding protein (CBP). In addition, recruitment of histone deacetylase 3 (HDAC3) by NF‐*κ*B results in local histone hypo‐acetylation and consequent decreased Nrf2‐ARE binding.^[^ ^43b^ ^]^ Our data indicated that the absence of Nrf2 both in vivo and in vitro could exacerbate NF‐*κ*B activity, leading to the increased expression of pro‐inflammatory biomarkers. Despite convincing evidence of functional interactions between the Nrf2 and NF‐*κ*B pathways, further investigations are needed to unravel the conditional and dynamic nature of this cross‐talk.

Although 30 min of ischemia has been widely utilized to create ischemia/reperfusion model in rodents,^[^ ^1b^ ^]^ the time period of ischemia is relatively short with less clinical significance. In addition, the time of hydrogel administration should be optimized in order to further enhance therapeutic efficacy. More importantly, pre‐clinical evaluations in large animal models are urgently needed to fully assess the translational potential of a dual‐function hydrogel developed in this study.

## 4 Conclusion

In summary, we designed a novel, injectable hydrogel with shear‐thinning properties for the treatment of MI. The hydrogel demonstrates a ROS‐responsive NO‐releasing function; therefore, it could effectively regulate local ROS/NO disequilibrium after I/R injury. In a mouse model, the CS‐B‐NO hydrogel was injected in situ into the ischemic myocardium and demonstrated the desired therapeutic efficacy, unlike hydrogels with only NO‐release or ROS‐scavenging properties. At the early stage of I/R injury, administration of the CS‐B‐NO hydrogel reduced myocyte apoptosis and alleviated the inflammatory response. Long‐term treatment with the CS‐B‐NO hydrogel promoted heart repair and improved cardiac function. Furthermore, we have provided mechanistic insight into the cardioprotective effect of the CS‐B‐NO hydrogel against I/R injury. On the one hand, the CS‐B‐NO hydrogel protected the heart against oxidative stress by enhancing Keap1 S‐nitrosylation to activate the Nrf2 signaling pathway. On the other hand, it reduced the upstream IKK/I*κ*B*α*‐mediated phosphorylation of NF‐*κ*B signaling, therefore inhibiting activation of the NF‐*κ*B signaling pathway and downstream pro‐inflammatory factor expression. The absence of Nrf2 attenuated the antioxidant capacities of the CS‐B‐NO hydrogel both in vitro and in vivo, suggesting that the cardioprotective effects of the CS‐B‐NO hydrogel were mediated by adaptive regulation of the Nrf2‐Keap1 defense system.

## 5 Experimental Section

Detailed methods are provided in the Supporting Information.

## Conflict of Interest

The authors declare no conflict of interest.

## Supporting information

Supporting Information

## Data Availability

The data that support the findings of this study are available from the corresponding author upon reasonable request.
