# Supplementary material for: An Injectable Dual‐Function Hydrogel Protects Against Myocardial Ischemia/Reperfusion Injury by Modulating ROS/NO Disequilibrium
Source: Adv Sci (Weinh). 2022 Mar 23;9(15):2105408. doi: 10.1002/advs.202105408 (PMC9130918; doi:10.1002/advs.202105408)
Supplement: Supplementary file 1 — Supporting Information [file ADVS-9-2105408-s001.pdf]

ADVANCED SCIENCE
Open Access

Supporting Information

for *Adv. Sci.*, DOI 10.1002/advs.202105408

An Injectable Dual-Function Hydrogel Protects Against Myocardial Ischemia/Reperfusion Injury by Modulating ROS/NO Disequilibrium

*Tian Hao, Meng Qian, Yating Zhang, Qi Liu, Adam C. Midgley, Yangping Liu, Yongzhe Che, Jingli Hou and Qiang Zhao**

Supporting Information

An injectable dual-function hydrogel protects against myocardial ischaemia/reperfusion injury by modulating ROS/NO disequilibrium

*Tian Hao, Meng Qian, Yating Zhang, Qi Liu, Adam C. Midgley, Yongzhe Che, Jingli Hou, Qiang Zhao**

# Hao T and Qian M equally contributed to this study.

Experimental section

Animal use and study approval: NFE2L2 knockout (Nrf2$^{-/-}$) mice on a C57BL/6 background were purchased from Shanghai Model Organisms Center. Wild-type (WT) C57BL/6 mice (weight 20-25 g; male; 8 weeks old) were purchased from SPF (Beijing) biotechnology Co., Ltd., Beijing, China. Animals were randomly grouped for treated and untreated controls. All experiments and animal procedures were approved by the Animal Experiments Ethical Committee of Nankai University and carried out in conformity with the Guide for Care and Use of Laboratory.

Synthesis of CS-B-NO: Chitosan (M_n = 50,000) with deacetylation degree of 90% was purchased from Haidebei bioengineering company (China). CS-NO was synthesized according to a published protocol.[1] All other chemicals and reagents were purchased from Sigma-Aldrich (China-mainland), Energy Chemical (China-mainland), and InnoChem (China-mainland), which were used directly without further purification. Silica gel (200-300 mesh) was used for column chromatography. ^{1}H NMR and ^{13}C NMR spectra were recorded on a Bruker Avance-400 FT nuclear magnetic resonance spectrometer. Chemical shifts were reported relative to the reference chemical shift of the NMR solvent. The following splitting abbreviations were used: s = singlet, d = doublet, dd = doublet doublet, t = triplet, m = multiplet.

CS-B-NO was first synthesized via two steps starting from CS (M_n = 50000). Briefly, Chitosan (0.5 g) was dispersed in 50 mL deionized water. Subsequently, 1 M hydrochloric acid (HCl) solution was added dropwise until the chitosan was completely dissolved, and then pH was adjusted to about 5 by adding 1 M aqueous NaOH. Certain amounts of EDC, DMAP, and pentynoic acid were added into chitosan solution. The reaction lasted for 24 h at room

temperature. The products were purified by consecutive dialysis for 3 days. The products of alkynyl-CS were finally obtained by lyophilization. Then alkynyl-CS (100 mg) was dissolved in 15 mL deionized water under the protection of N_2 atmosphere. B-NO, $CuSO_4$, and sodium ascorbate were added at the molar ratio 1:0.2:0.6. The reaction proceeded at room temperature for 24 h under N_2 atmosphere. After stopping the reaction, the products were dialyzed against deionized water for 3 days, and finally lyophilized.

Synthesis of CS-B: 4-(Bromomethyl) benzoic acid (34.4 g, 0.16 mol) was suspended in 2,3,3-trimethylindolenine (25 mL, 0.15 mol). The suspension was stirred at 120 °C for 12 hours under an argon atmosphere, and then allowed to cool to room temperature, and the resulting solid was collected by filtration. The solid was washed with chloroform and dried under reduced pressure to afford 1 as a pink solid. ^{1}H NMR (400 MHz, DMSO) δ 7.97 (d, *J* = 7.8 Hz, 2H), 7.88 (d, *J* = 7.2 Hz, 1H), 7.77 (d, *J* = 7.8 Hz, 1H), 7.65 – 7.48 (m, 4H), 5.92 (s, 2H), 2.97 (s, 3H), 1.62 (s, 6H). ^{13}C NMR (101 MHz, DMSO) δ 199.37, 167.22, 142.40, 141.55, 137.36, 131.58, 131.47, 131.17, 130.57, 130.06, 129.46, 129.32, 128.11, 124.19, 116.37, 55.11, 50.98, 22.74, 15.40 (Figure S4, Supporting Information).

Into a flask attached with Dean-Stark trap and a condenser were added compound 1 (14.43 g, 39.83 mmol), 2-chloro-1-formyl-3-(hydroxymethylene) cyclohex-1-ene (3.42 g, 20 mmol), n-butanol (300 mL), and benzene (30 mL). The mixture was refluxed for 12 h to give dark green solution. Solvents were removed on a rotavapor. Further chromatography on a silica gel column with gradient DCM/MeOH solvent system led to 8 as dark green solid. ^{1}H NMR (400 MHz, DMSO) δ 12.63 (s, 2H), 8.26 (d, *J* = 14.0 Hz, 2H), 7.94 (d, *J* = 8.2 Hz, 4H), 7.69 (d, *J* = 4.0 Hz, 2H), 7.43 – 7.37 (m, 8H), 7.31 (dd, *J* = 8.2, 4.0 Hz, 2H), 6.37 (d, *J* = 14.0 Hz, 2H), 5.62 (s, 4H), 1.91 (s, 4H), 1.73 (s, 14H). ^{13}C NMR (101 MHz, DMSO) δ 173.42, 172.47, 167.57, 167.37, 161.07, 149.30, 144.06, 142.77, 141.41, 140.50, 130.78, 130.49, 130.05, 127.58, 127.30, 127.15, 112.11, 105.95, 102.83, 75.41, 49.65, 49.07, 47.40, 45.20, 44.23, 30.31, 28.10, 26.20, 21.53 (Figure S5, Supporting Information).

To a solution of resorcinol (1.1 g, 10.0 mmol), CH_3CN, and K_2CO_3 (200 mg, 15.0 mmol) were mixed, and the mixture was stirred at 60 °C for 10 min. Subsequently, compound 2 (200 mg, 10.0 mmol) was dissolved in CH_3CN (10 mL) and added dropwise. The reaction mixture was allowed to stir at 60 °C. The crude product was then purified using flash chromatography on silica gel (CH_2Cl_2/MeOH as an eluent) to afford 3 as a green solid. ^{1}H NMR (400 MHz, MeOD) δ 8.74 (d, *J* = 12.0 Hz, 1H), 8.05 (d, *J* = 8.0 Hz, 2H), 7.67 (d, *J* = 8.0 Hz, 1H), 7.56 (s, 1H), 7.51 – 7.33 (m, 6H), 6.97 – 6.71 (m, 2H), 6.37 (d, *J* = 12.0 Hz, 1H), 5.63 (s, 2H), 2.76 (d,

J = 5.6 Hz, 2H), 2.56 (d, J = 5.6 Hz, 2H), 1.87 (s, 8H). ^{13}C NMR (101 MHz, DMSO) δ 177.09, 167.32, 162.87, 162.71, 154.87, 142.37, 141.90, 139.99, 136.79, 130.91, 130.55, 130.03, 127.33, 127.14, 115.15, 103.44, 102.47, 50.53, 30.31, 28.62, 28.28, 24.04, 20.37 (Figure S6, Supporting Information).

To a stirred solution of compound 3 (400 mg, 10.0 mmol), CH_3CN, and K_2CO_3 (200 mg, 15.0 mmol) were mixed, and the mixture was stirred at 40 °C for 30 min. Subsequently, 4-(Bromomethyl) benzeneboronic acid pinacol ester (200 mg, 10.0 mmol) was added dropwise. The reaction mixture was allowed to stir at 40 °C, and then the crude product was diluted with dichloromethane. The crude product was then purified using flash chromatography on silica gel (CH_2Cl_2/MeOH as an eluent) to afford 4 as a blue solid. ^{1}H NMR (400 MHz, $CDCl_3$) δ 8.36 (d, J = 14.2 Hz, 1H), 7.99 (d, J = 8.0 Hz, 2H), 7.73 (d, J = 7.8 Hz, 2H), 7.40 (d, J = 7.2 Hz, 1H), 7.36 – 7.26 (m, 5H), 7.22 (dd, J = 14.0, 6.4 Hz, 3H), 7.13 (d, J = 13.2 Hz, 2H), 7.02 (d, J = 7.8 Hz, 1H), 6.04 (d, J = 14.2 Hz, 1H), 5.49 (s, 2H), 5.27 (s, 2H), 2.56 (s, 2H), 2.40 (s, 2H), 1.74 (s, 8H), 1.25 (s, 12H). ^{13}C NMR (101 MHz, MeOD) δ 176.76, 165.62, 163.59, 163.02, 155.10, 145.13, 142.15, 141.31, 139.65, 139.22, 136.96, 134.58, 130.20, 130.00, 129.38, 128.85, 126.81, 126.54, 126.38, 126.16, 122.51, 115.27, 115.19, 111.75, 102.00, 101.61, 83.78, 77.82, 72.47, 66.31, 63.04, 62.91, 34.41, 31.67, 29.35, 28.37, 27.55, 27.25, 25.21, 23.81, 23.64, 22.71, 22.34, 20.16 (Figure S7, Supporting Information).

Chitosan (0.1 g) was dispersed in 50 mL deionized water. Subsequently, 1 M HCl was added dropwise until the chitosan was completely dissolved, and then pH was adjusted to about 5 by adding 1 M aqueous NaOH. Certain amounts of EDC, DMAP, and compound 4 were added into chitosan solution. The reaction lasted for 24 h at room temperature. The products were purified by consecutive dialysis for 3 days. The products of CS-B were finally obtained by lyophilization (Figure S8, Supporting Information).

Rheological properties and degradation: CS-B-NO was dissolved in saline at concentrations of 1 and 2 wt% to prepare CS-B-NO hydrogels. The rheological properties of the hydrogels were investigated by a dynamic rheology method using a Bohlin Advanced Rheometer with plate geometry at 25 °C. The hydrogel was added onto the plate, the upper plate was lowered to a measurement gap of 500 μm. A time sweep test with angular frequency of 1.0 rad/s and a strain of 2.0% were used for measurement. The viscosity of the hydrogel was measured with angular frequency of 1.0 rad/s and strains from 0.001% to 100%. The degradation profiles of CS-B-NO hydrogels (1 wt%) were performed by incubation with PBS with and without

addition of H_2O_2 (100 μM) at 37 ℃. Residual weight of the hydrogel was measured at different intervals after lyophilization.

Measurement of NO release: The NO releasing profile was determined by Griess kit assay. In brief, 5 mg of CS-B-NO was put into 5 mL of PBS buffer (pH 7.4) with different concentrations of H_2O_2 (10 mM, 1 mM, 100 μM, or 10 μM). At pre-determined time interval, 50 mL solution was transferred into 96-well plate, and 50 mL Griess I and 50 mL Griess II were added thereafter. The azo compound of purple color was formed and the absorbance was measured at wavelength of 540 nm using an iMark microplate reader (BIORAD).

In vitro NO and H_2O_2 detection: After stimulated with H_2O_2 for 24 hours, H9C2 cells were added into cell-culture dish pre-coated with 500 μL of hydrogel (CS-B, CS-NO or CS-B-NO). The production of H_2O_2 and NO in cell culture medium of different groups were detected 24 hours later. The level of H_2O_2 in supernatant was measured by using Hydrogen Peroxide Assay Kit (Beyotime Institute of Biotechnology, S0038) according to the manufacturer's instructions. The absorbance was measured at 560 nm (Synergy 4, BioTek Instruments). The level of NO was assessed by using chemiluminescence NO analyzer (NOA) (Seivers 280i, Boulder, CO). In brief, the samples were immersed in 5mL of Vanadium (III) chloride (50 mM). The generated NO gas was diffused in the test solution and transported to NO analyzer by a stream of N_2 (g). The calculation of the generated NO was based on the calibration curves of the NOA, which was described in detail before.[2]

H9C2 cells after different treatment were stained with MitoSOX Red (5 μM) (Yeasen Biotech Co.Ltd., 40778ES50) and DAF-FM DA (5 μM) (Beyotime Institute of Biotechnology, S0019), respectively. The mean fluorescence intensities were measured to quantify the superoxide and NO generation.

In vitro $ONOO^-$ detection: After stimulated with H_2O_2 for 24 hours, H9C2 cells were treated with CS-B, CS-NO, and CS-B-NO hydrogel as mentioned before. H9C2 cells were stained with DAX-J2™ PON Green 99 (AAT Bioquest, 16316), and mean fluorescence intensities were measured to quantify the peroxynitrite ($ONOO^-$) generation.

Murine myocardial I/R model: Surgical induction of cardiac ischaemia/reperfusion was performed on C57BL/6 mice (male, 8-weeks old) as previously described with some modification.[3] Briefly, all mice were anesthetized with 2% isoflurane. Mice were ventilated with a mechanical ventilation (Hallowell EMC Microvent I) setting at 110 breaths per minute with a tidal volume of 0.2-0.3 mL. The third intercostal space over the left chest and the heart

were exposed. After left thoracotomy, the left anterior descending coronary artery was ligated with a 6-0 silk ligature tied with a slip knot. The left main descending coronary artery at a site of about 3 mm from its origin was sutured and tied with a slip knot. Cardiac ischaemia was confirmed by the presence of myocardial blanching in the perfusion bed. Thirty minutes after ischaemia, the slip knot was released by pulling the outside end of the suture smoothly to re-perfuse the myocardium. Immediately after reperfusion, 30 μL of polymer solution (CS, CS-B, CS-NO or CS-B-NO) was injected into the myocardium. The concentration kept at 1 wt% except for CS-NO (0.5 wt%) in order to obtain similar NO releasing amount. Acidified CS (chitosan hydrochloride) was utilized due to its good solubility in saline. Chest cavity was closed to restore negative pressure to prevent pneumothorax. Mice of I/R group only received ischaemia/reperfusion without hydrogel injection, while sham operated mice only experienced thoracotomy without ischaemia/reperfusion surgery or hydrogel injection.

In vivo NIR imaging: Mice after ischaemia/reperfusion surgery were administrated with above mentioned hydrogels that containing 1mM H_2O_2-sensitive NIR probe via in situ myocardial injection. One day after surgery, the mice were slightly anesthetized. H_2O_2 specific signal was detected by using CRI Maestro noninvasive fluorescence imaging system. All images were acquired through 640–820 nm range in 10 nm steps using the "orange" filter with excitation at 605 nm (exposure time 1000 ms; n=3 animals per group).

In vivo NO release detection: Electron paramagnetic resonance was performed to detect the level of NO in heart tissues after different treatments as previously reported with some modification.[4] Briefly, at one day after surgery, mice were slightly anesthetized in box with isoflurane, and injected subcutaneously with DETC sodium salt (500 mg/kg, Sigma-Aldrich) diluted in distilled deionized water (250 mM). Five minutes later, ammonium ferrous sulfate (50 mM) citrate solution (250 mM) was injected subcutaneously on the other side (2 mL/kg). After one hour, heart samples were collected and frozen in liquid nitrogen immediately. The frozen tissues were homogenized in homogenate tubes and extracted with ethyl acetate (200 μL) immediately. The ethyl acetate extract was concentrated with nitrogen and transferred to a 50 μL capillary, then the X-band EPR was measured at room temperature (23±1 ℃). The following acquisition parameters were used: modulation frequency, 100 KHz; microwave power, 10 mW; modulation amplitude, 2 G; number of scans, 60. The double integrated area of EPR spectra was calibrated into concentrations of NO-Fe(MGD)$_2$ using TEMPO as a standard. EPR spectral simulation was conducted by the WINSIM program.

In vivo H_2O_2 level detection: After the heart ischaemia/reperfusion of 24 hours, mice were sacrificed. The hearts were excised and homogenized. The level of H_2O_2 in heart tissues was measured with 10% homogenates by using Hydrogen Peroxide Assay Kit (Beyotime Institute of Biotechnology, S0038) according to the manufacturer's instructions. The absorbance was measured at 560 nm (Synergy 4, BioTek Instruments).

Cell Culture: Primary mouse cardiomyocytes were isolated from pups of C57 mice within the birth of 3 days according to a reported protocol.[5]The cell suspension was transferred to the Laminin coated (Sigma) T75 flask and incubated overnight. The culture medium was replaced by DMEM High Glucose with 1% ITS and 1% PS after 24 hours of myocardial cell attachment.

H9C2 cells were maintained in DMEM (GIBICO, USA) medium supplemented with 10% fetal bovine serum (FBS, GIBCO, USA) and 1% penicillin-streptomycin solution (GIBCO, USA) and placed in a cell culture chamber containing 5% CO_2 at 37 °C. To knockdown Nrf2 *in vitro*, the specific Nrf2 inhibitor (ML385, HY-100523, MCE) was applied to the experiment. The procedure was performed according to the manufacturer's instructions, and the Nrf2-knockdown H9C2 cells were expanded for subsequent experiments.

Flow cytometric analysis: Single-cell suspension was prepared according to the protocol described previously.[6] Briefly, the mice were deeply anesthetized and the chest was opened to expose heart. Cardiac perfusion was performed immediately with precooled PBS to remove blood cells. The hearts were dissected, cut into pieces with scissors, and enzymatically digested in DMEM High Glucose (Gibco) with a cocktail of 1 mg/mL type II collagenase (Invitrogen) and 100 U/mL DNase I (Sigma-Aldrich) for 1.5 hours at 37 °C with 220 rpm agitation. After complete digestion, the mixture of tissues and cells were passed through a 70-μm cell strainer (BD Falcon, NJ, USA). The obtained cells were centrifuged at 2000 rpm for 10 minutes, then the cells were isolated and washed with DMEM high glucose cell culture medium for further analysis.

ROS-positive cells in cardiac tissue were stained by a ROS detection kit (Nanjing Jiancheng Bioengineering Institute, China, E004-1-1) after single cells were collected. The obtained results were expressed as the percentage of ROS-positive cells in cardiac tissue. The results of flow cytometric were analyzed using FlowJo software.

Infarct size assessment: After myocardial ischaemia for 30 minutes and reperfusion for 2 days, thoracotomy was performed. The heart was quickly excised after quick freezing for 15 min, and sliced at 1-mm thickness. Afterward, the sections were incubated with 1.5% 2,3,5-

triphenyltetrazolium chloride (TTC; Sigma-Aldrich) solution at 37 °C in an incubator for 15-30 minutes and then with 4% formaldehyde solution for 2 hours. The normal myocardial tissue was red, while the ischaemic myocardium is white. The size of the infarcted myocardium, which was white or pale, was measured by Image J software.

Myocardial apoptosis: After myocardial ischaemia for 30 minutes and reperfusion for 24 hours, TUNEL staining was performed. Briefly, thoracotomy was performed to expose the heart that were subsequently fixed with trans-cardiac perfusion of saline and immersed in 4% paraformaldehyde over 24 hours. The heart samples were fixed with 4% PFA, immersed in 30% sucrose solution over 48 hours, and embedded with OCT. The samples were cut into 5-μm thick sections and stored at -20 °C. The cell apoptosis was detected as instructed in the protocol accompanying Dead End™ Fluorometric TUNEL System (Promega, G3250). Latterly, the sections were co-stained with cardiomyocyte marker α-SA (ab9465) at 4 °C overnight. Goat anti-mouse IgG antibody was applied for 1.5 hours at room temperature as a secondary antibody. Cell nuclei were stained with DAPI. Green fluorescence-labeled apoptotic cells were counted with Image J.

The protection of hydrogel on cardiomyocytes apoptosis assessment *in vitro* was performed with neonatal mice cardiomyocytes treated with different hydrogels after 200 mM H_2O_2 stimulated 24 hours to induce cell apoptosis. An Annexin V/PI assay kit (Solarbio) was used to measure the amount of cardiomyocytes apoptosis.

Myocardial injury measurement: The blood of the retro-orbital plexus was collected at 5 days after myocardial ischaemia/reperfusion injury. The supernatant was removed after centrifugation and the levels of cTnT and LDH (indicators of myocardial injury) were analyzed. cTnT concentration was determined using a Mouse cTnT ELISA Kit (cat.: E-EL-M1801c, Elabscience), and the concentration of LDH was measured using an LDH detection Kit (cat.: BC0685, Solarbio), according to the manufacturer's instructions. Following the addition of stop solution, the absorbance was measured at 450 nm (Synergy 4, BioTek Instruments).

Echocardiographic analyses of cardiac function: Transthoracic echocardiograph was performed with a Vevo 2100 Imaging System (Fuji Film Visual Sonics, Inc. Toronto, Canada) equipped with an MS-400 imaging transducer. The baseline of mice cardiac function was measured at 3 days before surgery. The cardiac function was analyzed at days 1,7,14, and 28 after I/R surgery with different treatments as reported previously. Mice were slightly anesthetized in box with isoflurane. Their limbs were fixed in a supine position on the echo

mat and the chest hair was removed by depilating cream. Then the mice were anesthetized with inhalation of isoflurane (0.5 to 1%) mixed with oxygen to maintain the heart rate at approximately 500-600, and the M-mode echocardiography was performed. The left ventricular internal diameter at end-diastole (LVIDd) and systole (LVIDs) were obtained through measuring the long axis and the short axis. Accordingly, the cardiac parameters of LV-ejection fraction (LV-EF), LV-fractional shortening (LV-FS), LV end-diastole volume (LV-EDV) and LV end-systole volume (LV-ESV) were determined. The echocardiography results were obtained in a double-blind way.

Histological analysis: At indicated time points, mice were anesthetized via intraperitoneal injection of 4% chloral hydrate (100 μL/10g) and thoracotomy was performed. The hearts were fixed with trans-cardiac perfusion of saline and immersed in 4% paraformaldehyde over 24 hours. The heart tissue samples were dehydrated with gradient alcohol and xylene and then embedded in paraffin blocks and cut into 5 μm thickness sections. The frozen sections were treated in the same way as previously mentioned.

The paraffin-sections were stained with Masson trichrome, Hematoxylin-Eosin (H&E), and Sirius Red following a standard protocol. Immunofluorescence staining was performed on the paraffin-embedded sections of the heart tissue samples. After deparaffinization and heat-mediated antigen retrieval in citrate solution, the samples were washed with PBS for three times and incubated with blocking serum which was used to avoid nonspecific binding at room temperature for 30 minutes. The sections were incubated with specific antibodies diluted in goat serum at 4 °C overnight. On the second day, the sections were rewarmed at room temperature for 1 hour and washed with PBS three times. Afterwards, the sections were incubated with Alexa-Fluor-coupled secondary antibodies for 2 hours at room temperature. After washing with PBS, the sections were counterstained with DAPI-containing fluoromount-G (SouthernBiotech, 0100-20) and coverslipped.

DHE staining: The production of superoxide was detected by dihydroethidium (DHE, Beyotime Institute of Biotechnology, S0063) staining. Briefly, the frozen heart sections were incubated with DHE (2 μM) for 30 min.

Western Blotting: Heart tissue and cell samples were homogenized in RIPA buffer (Solarbio, Beijing, China) with the supplement of 1% PMSF (Solarbio, Beijing, China).The protein concentration was determined by a BCA protein assay kit (Solarbio, Beijing, China).The protein level of BCL2 (sc-492), Bax (50599-2-Ig), Bad (A19595), Caspase3 (ab4051), Cleaved Caspase3 (#9664s), Nrf2 (ab89443), Keap1 (ab139729), SOD1 (bs-10216R), SOD2

(ab13533), HO-1 (w102400), HO-2 (bs-1238R), NQO-1 (A19586), NF-κB (sc-109), IKK (sc-7607), p-IKK (#2694), IκBα (ab32518), p-IκBα (wl02495), IL-10 (wl03088), Arg-1 (16001-1-AP), CD206 (ab64693), IL-1β (w100891), IL-6 (wl02138), TNF-α (sc-52746), and iNOS (ab15323) were detected with specific primary antibodies. The level of β-actin (sc-47778) expression was used as an internal control.

Total proteins were separated by SDS-polyacrylamide electrophoresis gel, and transferred to PVDF membranes (Millipore, Merck, Darmstadt, Germany). The membranes were blocked with 5% non-fat milk in Tris-buffered saline with 0.1% Tween 20 (TBST) for 2 h at room temperature, and incubated with the indicated primary antibodies at 4 °C overnight. On the second day, the membranes were washed six times for 30 min each with TBST, and incubated with 1:2000 dilutions of horseradish peroxidase-coupled secondary antibodies for 2 h at room temperature. Blots were once again washed six times for 5 min each in TBST and detected by ECL reagent. The obtained images were analyzed using Image J.

Quantitative real-time PCR: Total RNA samples from the cells were prepared using TRIeasy™ Total RNA Extraction Reagent (Yeasen Biotech Co.Ltd., 10606ES60) according to the manufacturer's instructions. The murine heart tissue samples were collected at the indicated time points after I/R surgery.

The tissue samples were dissected at the border zone of the left ventricular and frozen in liquid nitrogen immediately. Afterwards, the total RNA was extracted with Trizol reagent as mentioned previously. The concentration of the RNA was measured with a NanoDrop spectrophometer (NanoDrop Technologies). The complementary cDNA was synthesized using a first-strand cDNA synthesis kit (Yeasen Biotech Co.Ltd., 11141ES60). Quantitative real-time PCR was performed on a CFX96 Real-Time PCR System (Bio-Rad, Hercules, CA) with SYBR Green-based real-time detection system (Yeasen Biotech Co.Ltd.,11203ES03). The relative gene expression of mRNA was expressed as $2^{-(\triangle\triangle CT)}$ and normalized to GAPDH as an internal control. Each reaction was performed in triplicate to obtain average value and the changes in relative gene expression normalized to the internal control levels were determined. The highly purified primers used in this experiment were commercially synthesized (Sangon Biotech Co. Ltd., Shanghai, China). The sequences of primers used in this experiment were summarized in Supplementary Table 2.

Detection of free thiol in vivo: The free thiol content of the heart tissues was determined by monobromobimane fluorescence as previously reported.[7] Briefly, one day after surgery, mice were anesthetized by I.P. injection of 4% chloral hydrate. The heart samples were collected

and frozen in liquid nitrogen immediately. The frozen heart tissues were homogenized with homogenate tubes. The mixture was passed through Sephadex G-25 spin columns and reacted with monobromobimane (50 μM) for 1 hour at room temperature. Fluorescence intensities were measured via a microplate reader with λ_{ex} = 390 nm and λ_{em} = 480 nm.

Biotin switch assay of S-Nitrosylation: A biotin switch assay was carried out with a S-Nitrosylated Protein Detection Kit (Cayman.10006518). Briefly, heart tissue or cell samples were digested into single-cell suspensions. Then the biotin labeled nitrosylated proteins were obtained by the kit. Finally, the proteins were purified by streptavidin-agarose beads and subjected to western blotting with the specific antibody.

Statistics: All data were presented as mean ± SEM from at least three independent experiments. Comparisons between two groups were performed by Student t-test and comparisons among more than two groups was performed by one-way or two-way ANOVA. Statistical analyses were performed with GraphPad Prism software 7.0, and a statistical significance was accepted at p value of less than 0.05.

Supplementary Tables

Table.S1. List of the antibodies and kits used in this study

Antibody specificity	Company	Cat. No.	Application	Dilution
Mouse antibody for Western Blot				
BCL2	Santa Cruz	sc-492	WB	1:500
Bax	proteintech	50599-2-Ig	WB	1:2000
Bad	Abclonal	A19595	WB	1:500
Caspase3	abcam	ab4051	WB	1:500
Cleaved Caspase3	Cell Signaling	#9664s	WB	1:1000
Nrf2	abcam	ab89443	WB	1:500
Keap1	abcam	ab139729	WB	1:500
SOD1	Bioss	bs-10216R	WB	1:500
SOD2	abcam	ab13533	WB	1:5000
HO-1	Wanleibio	w102400	WB	1:1000
HO-2	Bioss	bs-1238R	WB	1:500
NQO-1	Abclonal	A19586	WB	1:500
CAT	Affinity Biosciences	DF7545	WB	1:500
NF-κB	Santa Cruz	sc-109	WB	1:500
IKK	Santa Cruz	sc-7607	WB	1:500
p-IKK	Cell Signaling	#2694	WB	1:1000
IκBα	abcam	ab32518	WB	1:1000
p-IκBα	Wanleibio	wl02495	WB	1:1000
Arg-1	proteintech	16001-1-AP	WB	1:5000
IL-10	Wanleibio	wl03088	WB	1:1000
CD206	abcam	ab64693	WB	1:500
IL-1β	Wanleibio	w100891	WB	1:500
IL-6	Wanleibio	wl02138	WB	1:1000
TNFα	Santa Cruz	sc-52746	WB	1:500
iNOS	abcam	ab15323	WB	1:500
β-actin	Santa Cruz	sc-47778	WB	1:5000

Mouse antibody for IF				
α-SMA	abcam	ab5694	IF	1:100
CD31	BD Biosciences	BD557355	IF	1:100
α-actinin	abcam	ab9475	IF	1:100
Cardiac Troponin I	abcam	ab47003	IF	1:100
Nitrotyrosine	Invitrogen	A-21285	IF	1:100
iNOS	abcam	ab15323	IF	1:50
CD206	abcam	ab64693	IF	1:100
eNOS	abcam	ab76198	IF	1:100
Vimentin	abcam	ab92547	IF	1:100
Secondary antibody conjugated with fluorescence				
Goat anti-Rabbit Secondary Antibody-Alexa Fluor® 594	Invitrogen	A-11037	IF	1:2000
Goat anti-Rabbit Secondary Antibody-Alexa Fluor® 488	Invitrogen	A-11008	IF	1:2000
Goat anti-Mouse Secondary Antibody-Alexa Fluor® 594	Invitrogen	A-11032	IF	1:2000
Goat anti-Mouse Secondary Antibody-Alexa Fluor® 488	Invitrogen	A-11001	IF	1:2000
Goat anti-Rat Secondary Antibody-Alexa Fluor® 555	Invitrogen	A-21434	IF	1:2000
Mouse ELISA kit				
Mouse cTnT ELISA Kit	Elabscience	M1801c		
Other kits				
AnnexinV-FITC/PI	Solarbio	CA1020	Apoptosis	According to protocol
Collagen II	Solarbio	C8150	Tissue digestion	75U/mL
TTC	Sigma	T8877-5G	Masson staining	1.5%

LDH detection kit	Solarbio	BC0685	LDH detect	According to protocol
Hydrogen Peroxide Assay Kit	Beyotime	S0038	H_2O_2 detect	According to protocol
ROS detection kit	Nanjingjiancheng	E004-1-1	FACS	According to protocol
DeadEnd™ Fluorometric TUNEL System	Promega	G3250	Apoptosis	According to protocol
S-Nitrosylated Protein Detection Kit	Cayman	10006518	S-Nitrosylated Protein Detection	According to protocol
Pierce® Streptavidin Agarose Resins	Thermo Scientific	20347	Affinity purification	According to protocol
Dihydroethidium	Beyotime	S0063	Superoxide detect	According to protocol
ML385	MCE	HY-100523	Nrf2 inhibitor	According to protocol

Table.S2. Primers used for Real-time quantitative PCR (qPCR)

	Forward primer (5' to 3')	Reverse primer (5' to 3')
Nrf2	TTGGCAGAGACATTCCCATTTG	AAACTTGCTCCATGTCCTGCTCTA
HO-1	TGCAGGTGATGCTGACAGAGG	GGGATGAGCTAGTGCTGATCTGG
NQO1	CAGCCAATCAGCGTTCGGTA	CTTCATGGCGTAGTTGAATGATGTC
BCL2	CGACTTCTTCAGCATCAGGA	TGAGCCACAGGGAGGTTCT
Bax	CGGCGAATTGGAGATGAACTG	GCAAAGTAGAAGAGGGCAA
Caspase3	TGACTGGAAAGCCGAAACTC	GCAAGCCATCTCCTCATCA
IL10	TCTTGCACTACCAAAGCCACA	CCAGTCAGTAAGAGCAGGCA
Arg1	CAGAAGAATGGAAGAGTCAG	GGTGACTCCCTGCATATCTG
IL6	CCACTTCACAAGTCGGAGGCTTA	TGCAAGTGCATCATCGTTGTTC
IL-1β	TCCAGGATGAGGACATGAGCAC	GAACGTCACACACCAGCAGGTTA
TNF-α	ACTCCAGGCGGTGCCTATGT	GTGAGGGTCTGGGCCATAGAA
GAPDH	CGGAGTCAACGGATTTGGTCGTAT	GAAGATGGTGATGGGCTTCC

Supplementary Figures

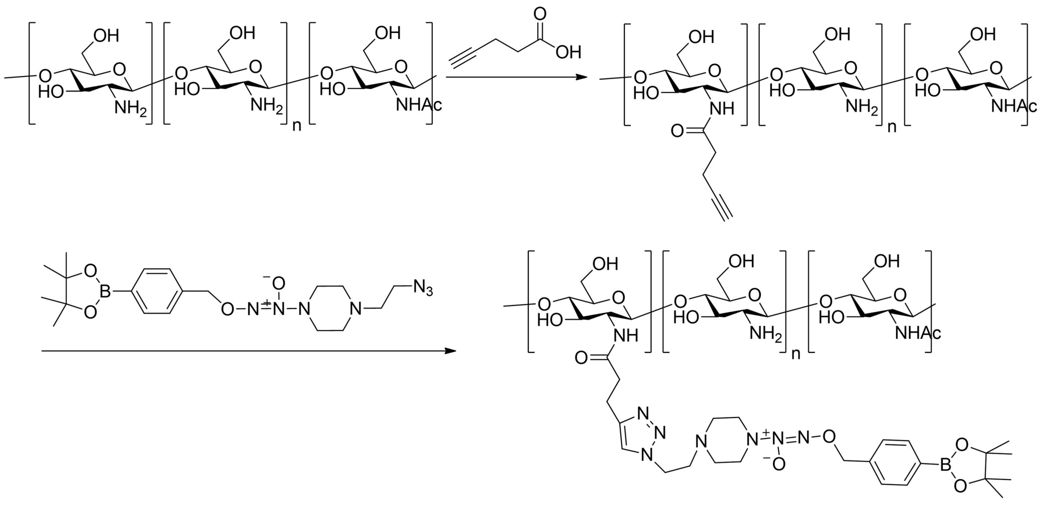

Figure S1. Synthesis of CS-B-NO.

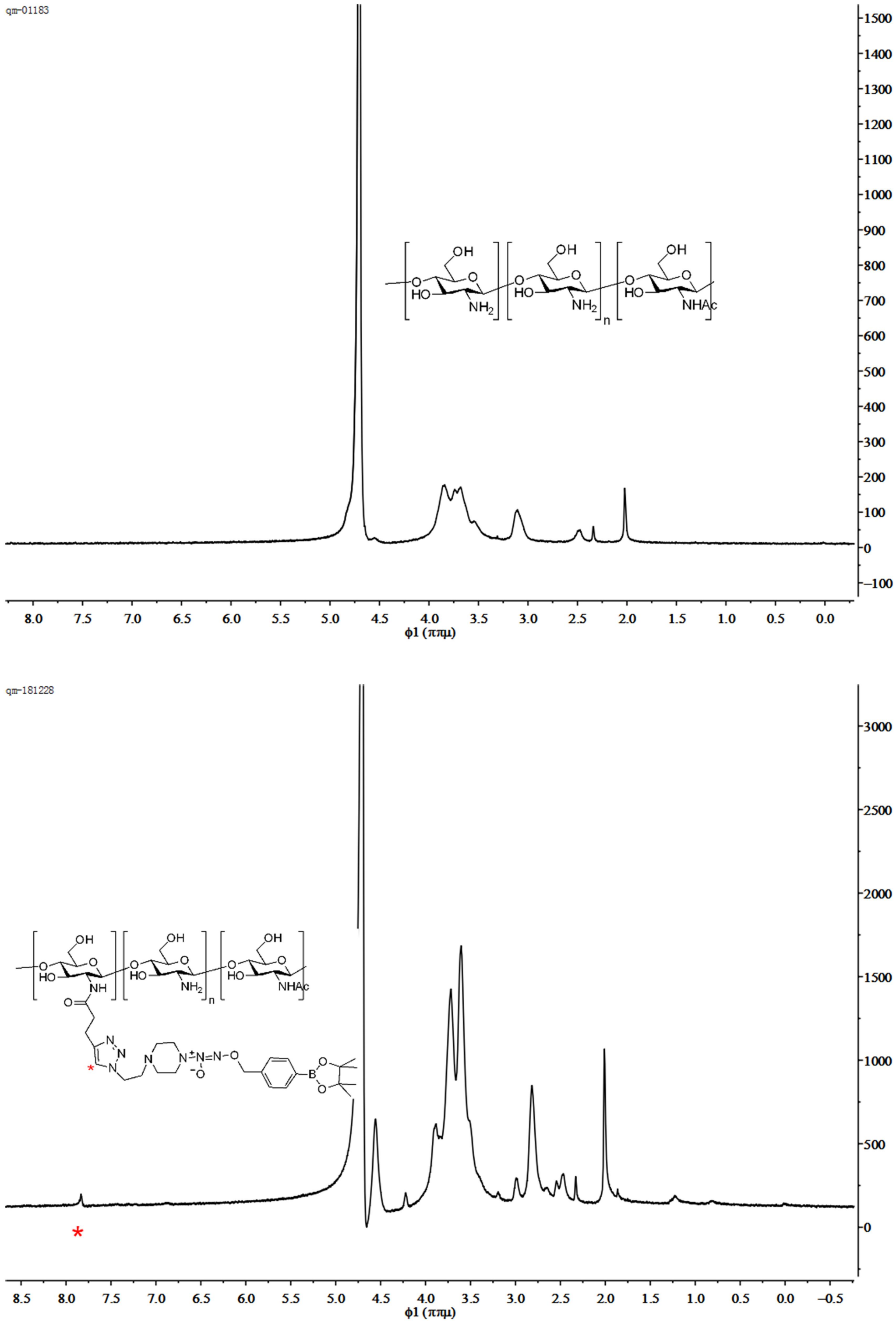

Figure S2. ^{1}H NMR spectra of CS and CS-B-NO.

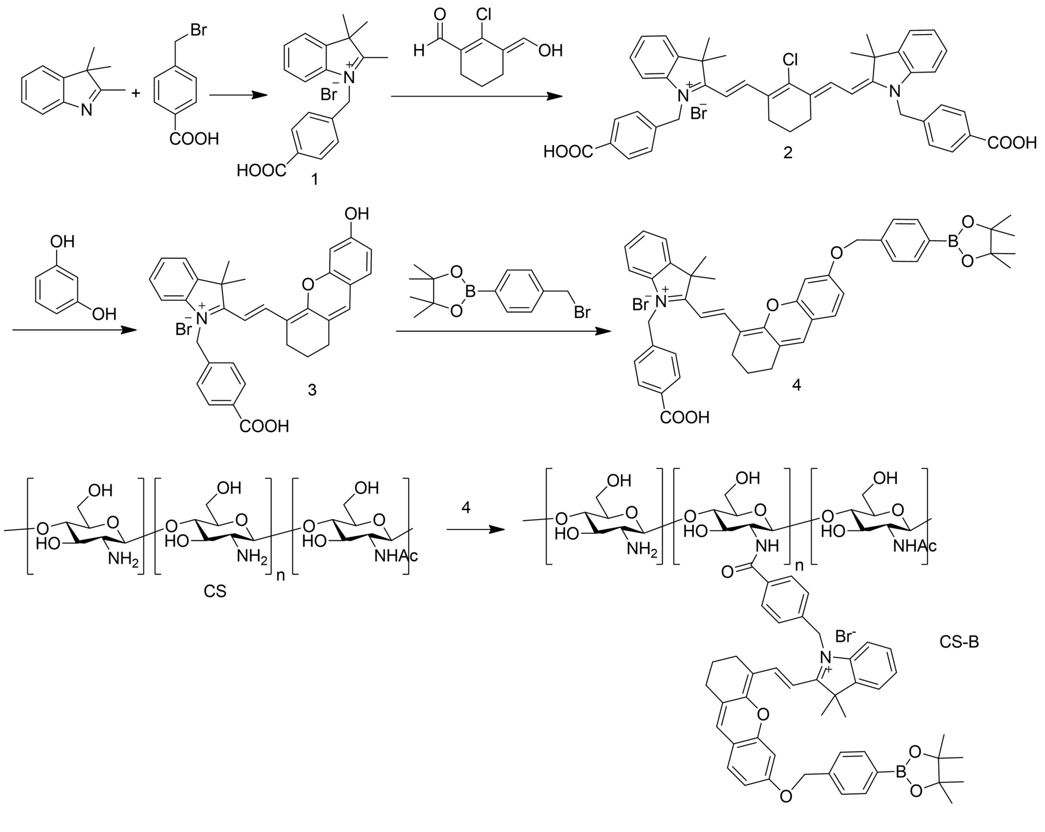

Figure S3. Synthesis of CS-B.

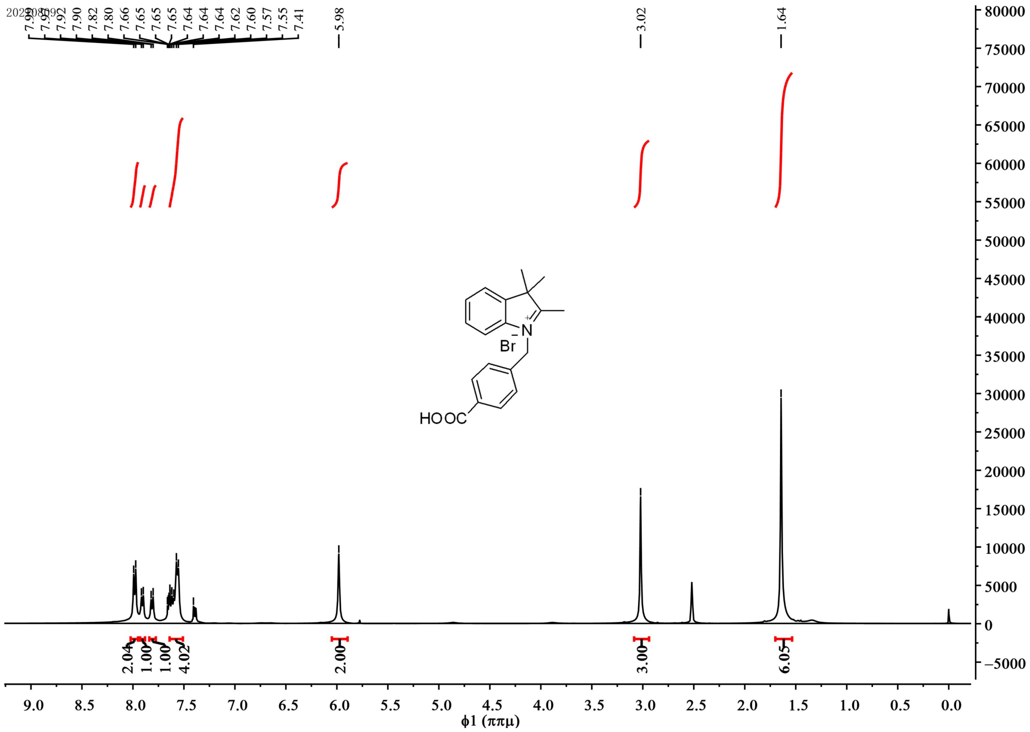

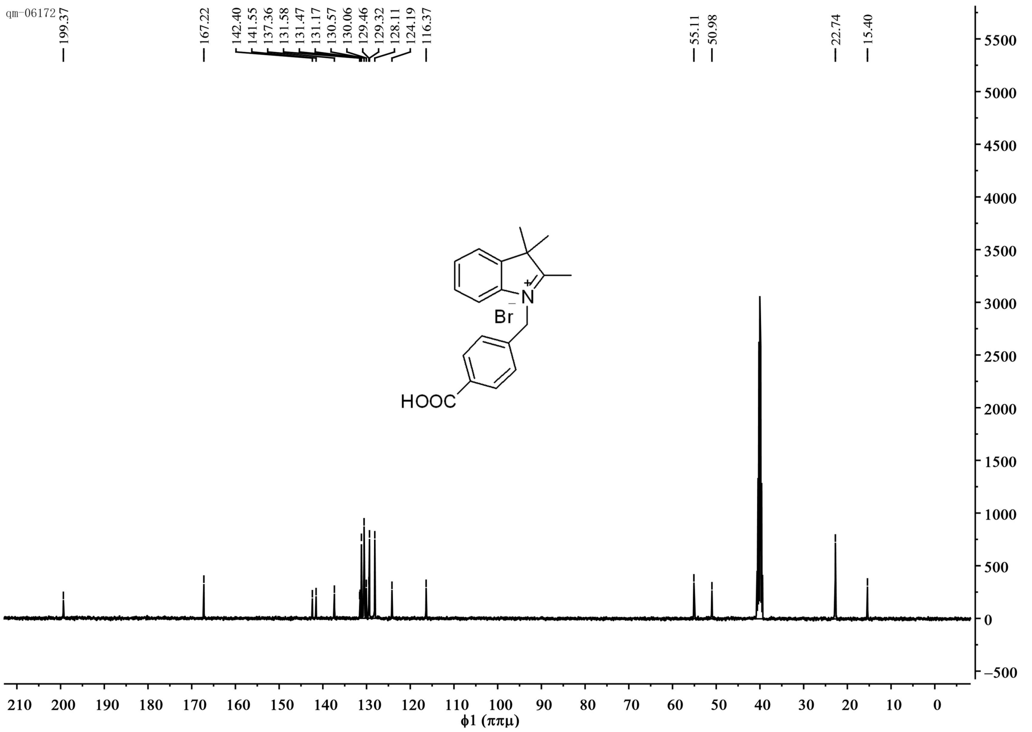

Figure S4. ^{1}H and ^{13}C NMR spectra of compound 1**.**

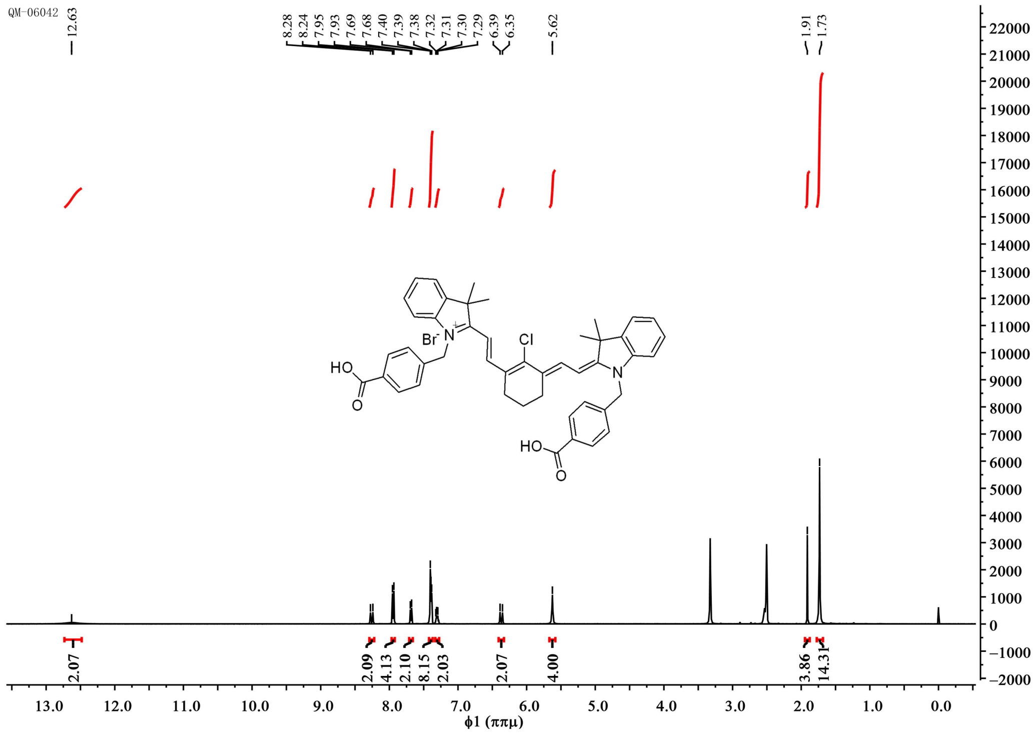

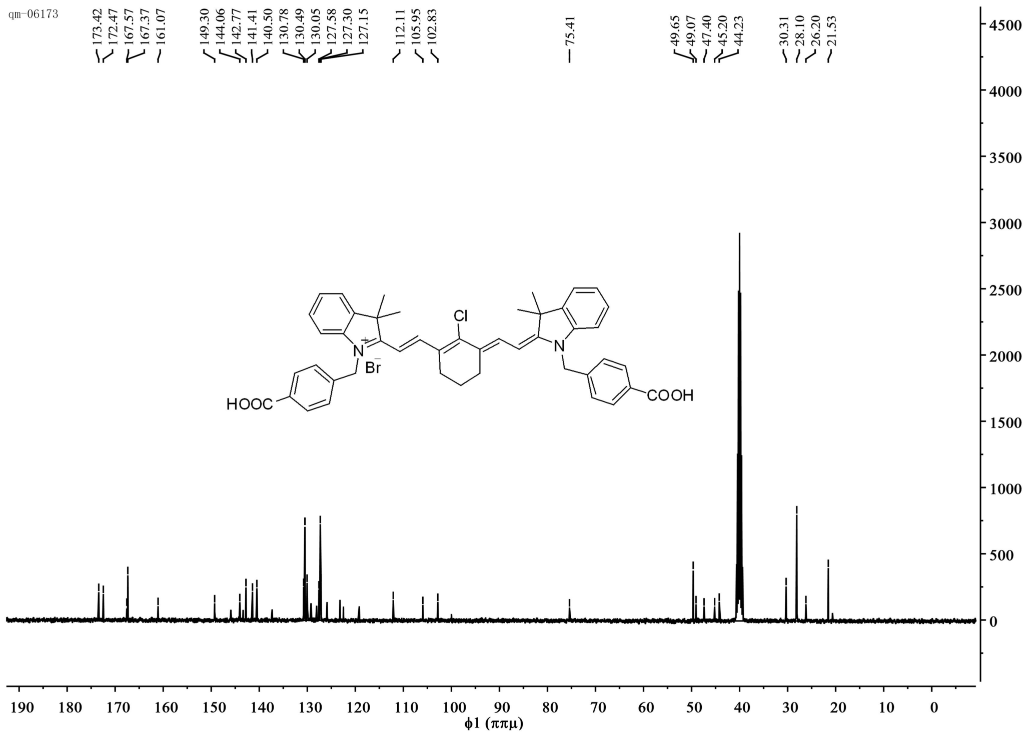

Figure S5. ^{1}H and ^{13}C NMR spectra of compound 2.

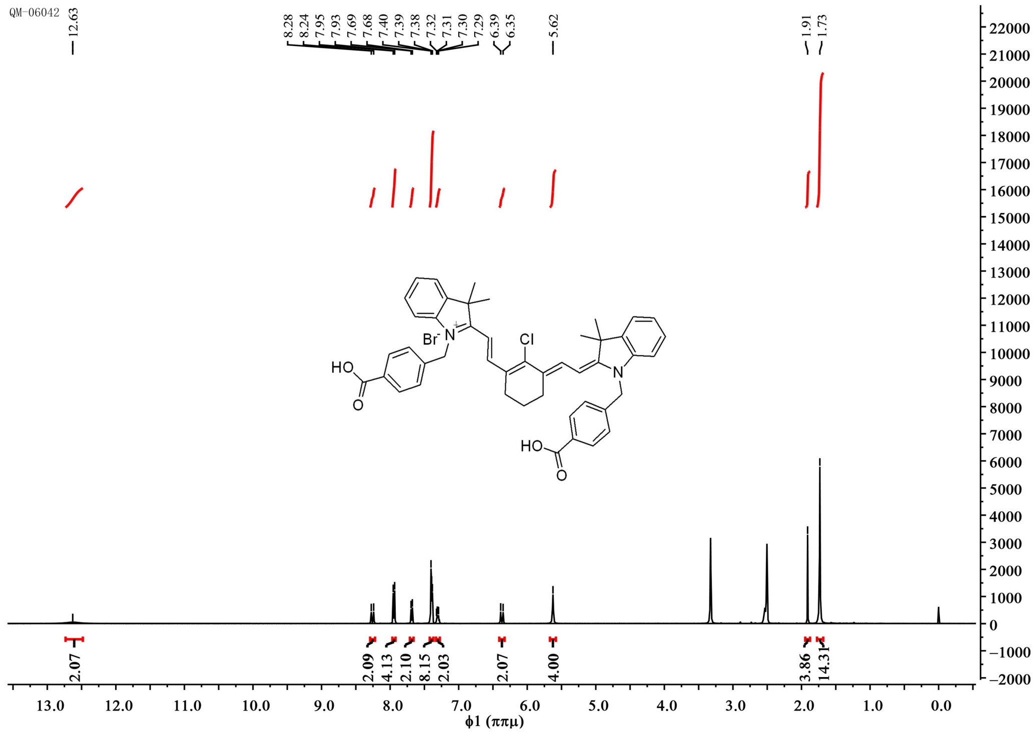

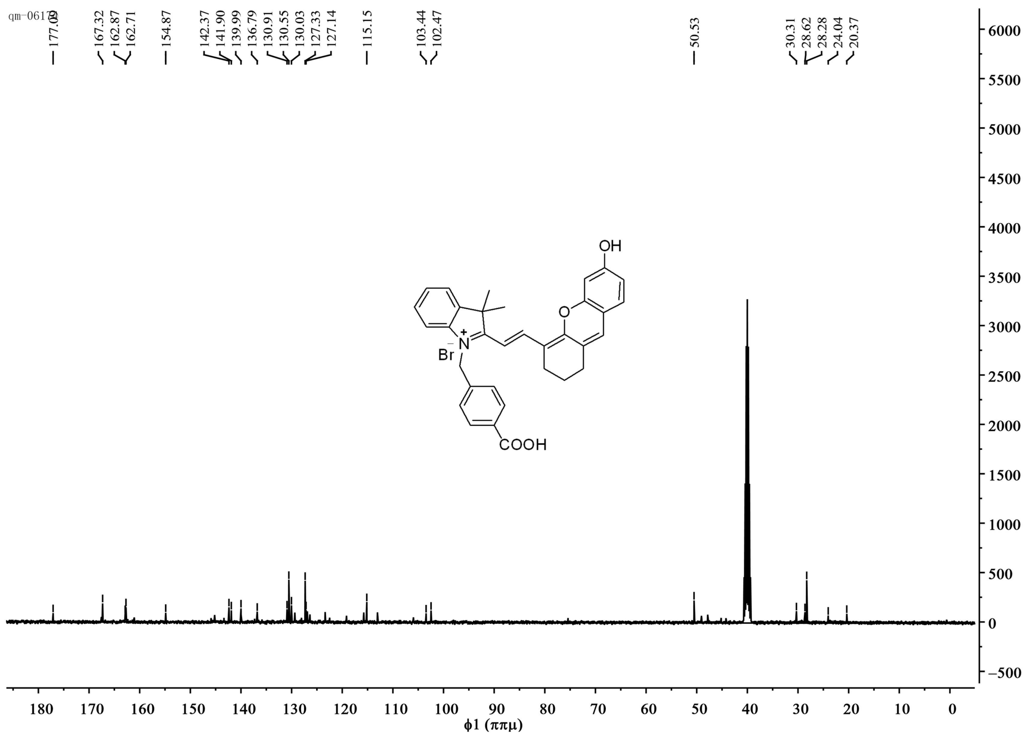

Figure S6. ^{1}H and ^{13}C NMR spectra of compound 3.

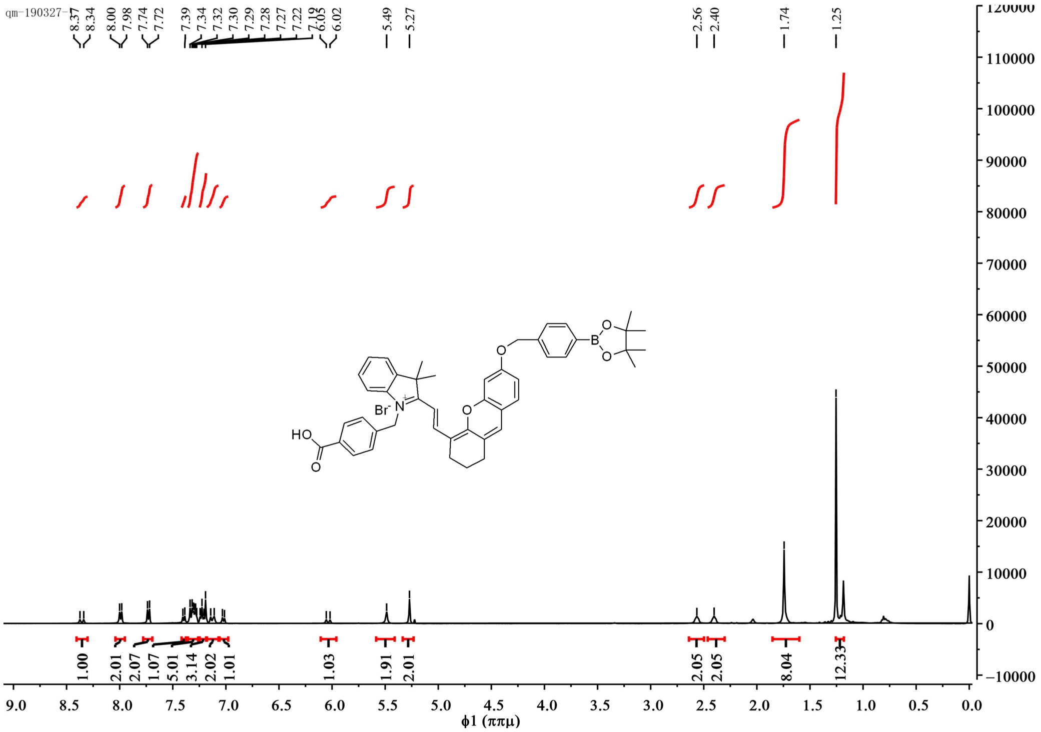

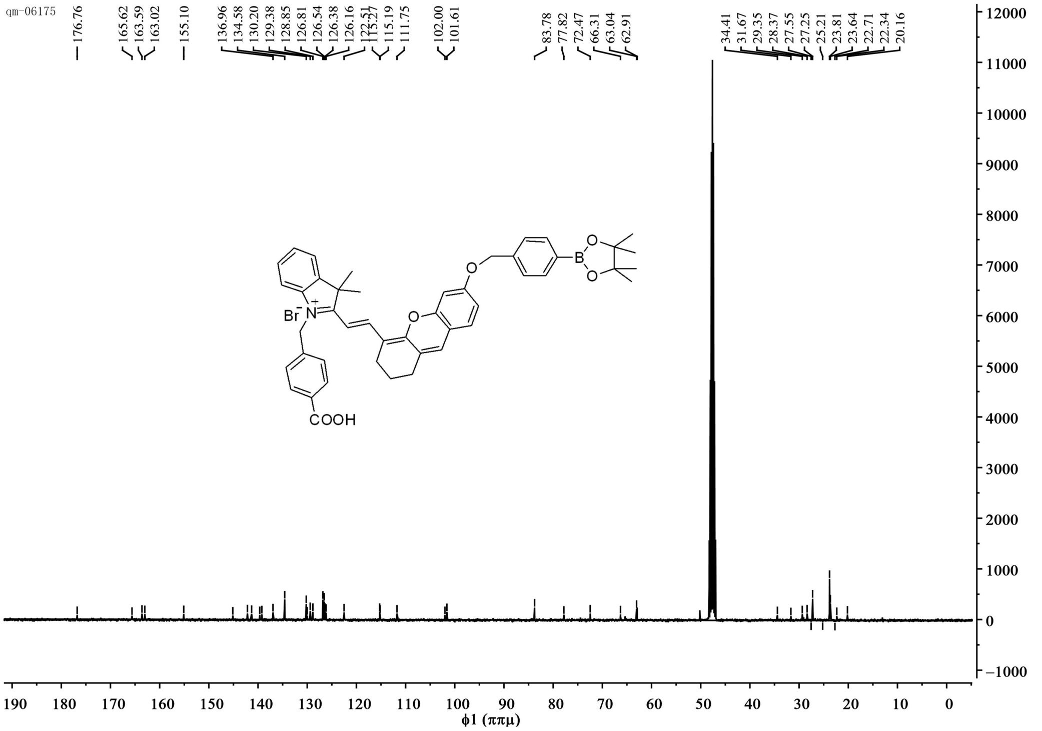

Figure S7. ^{1}H and ^{13}C NMR spectra of compound 4**.**

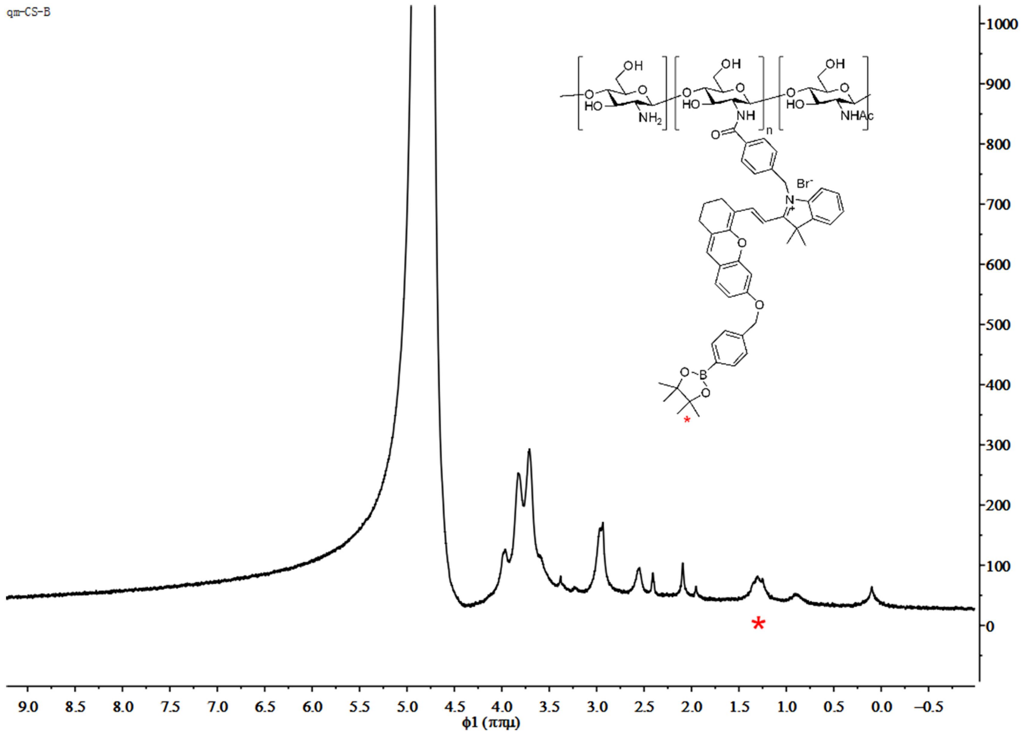

Figure S8. ^{1}H NMR spectra of CS-B.

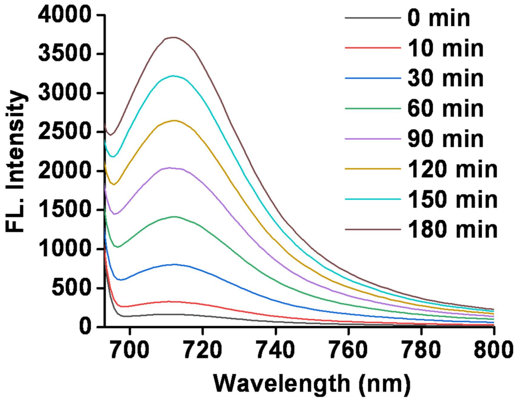

Figure S9. Relationship of the fluorescent density of the hydrogel with reaction time after treatment with 100 μM hydrogen peroxide.

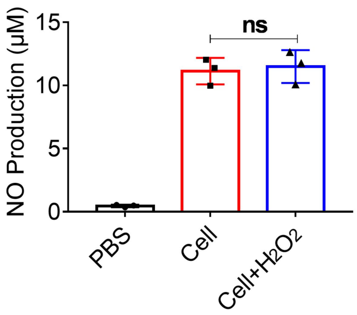

Figure S10. Release of NO from CS-NO after different treatments. Data are expressed as mean ± SEM, n=3 individual experiments. Significant difference was detected by unpaired student t-test.

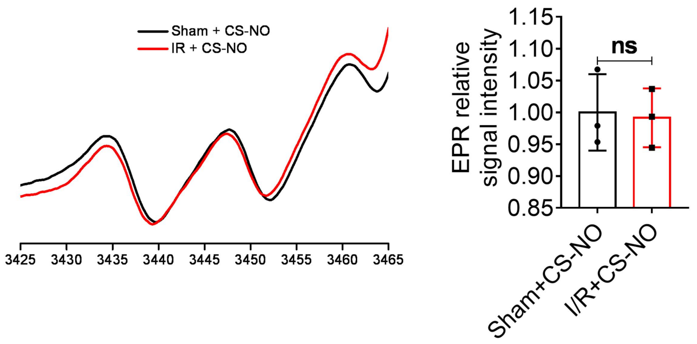

Figure S11. Nitric oxide level was detected by EPR. Representative EPR spectra reflecting NO generation from CS-NO hydrogels in Sham and I/R group. EPR relative signal intensity was calculated based on the peak height. Data are expressed as mean ± SEM, n=3 animals for each group. Significant difference was detected by unpaired student t-test.

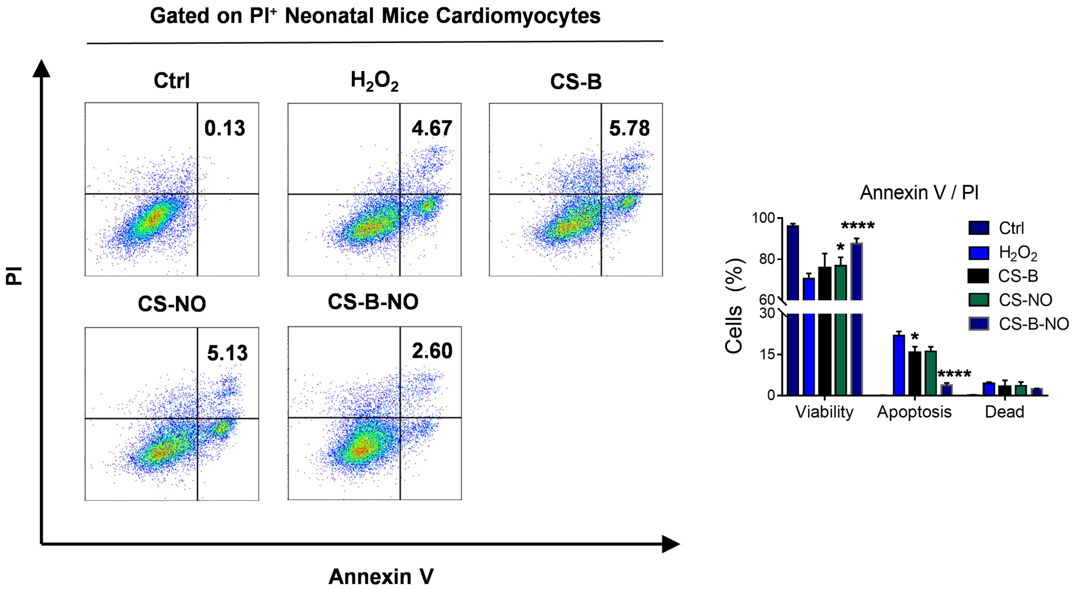

Figure S12. Effects of the CS-B-NO hydrogel on cell viability and apoptosis in neonatal mouse cardiomyocytes after H_2O_2 stimulation. Flow cytometry graphs showing cell viability and apoptotic populations in neonatal mouse cardiomyocytes by different treatments. Cells were treated with FITC-labeled Annexin V and PI staining (viability; apoptosis; dead cells). Mean percent values of cell viability, apoptotic and dead cells in neonatal mouse cardiomyocytes were calculated to assess the influence of the hydrogel treatment on cell viability and apoptosis. Data are expressed as mean ± SEM, n=3 individual experiments. Significant differences were detected by two-way ANOVA with Tukey's multiple comparisons test, *p<0.05,****p<0.0001 versus H_2O_2 stimulate group.

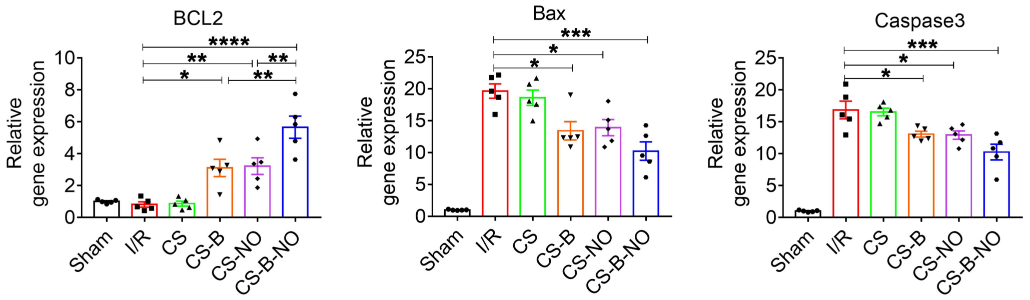

Figure S13. Effect of the CS-B-NO hydrogel on the expression of apoptosis-related genes. Gene expressions of apoptosis-related genes (BCL2, Bax and Caspase3) in mice hearts were detected 3 days after surgery by RT-PCR. Data are expressed as mean ± SEM, n=5 animals per group. Significant differences were detected by one-way ANOVA with Tukey's multiple comparisons test, *p<0.05 , **p<0.01, ***p<0.001, ****p<0.0001.

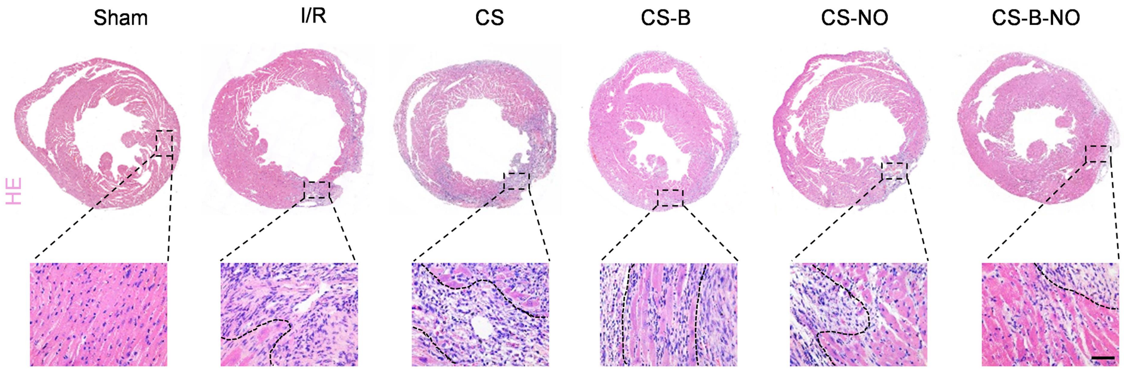

Figure S14. CS-B-NO hydrogel improved cardiac morphology. H&E (scale bar=50 μm) staining was performed and myocardium fibrosis was marked by the dotted line.

.

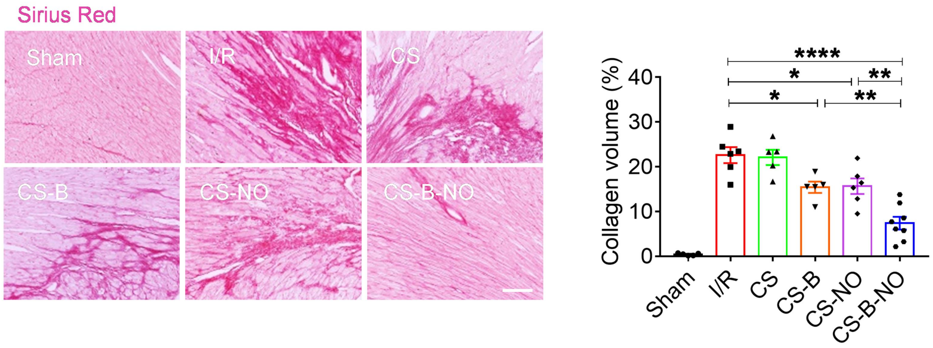

Figure S15. CS-B-NO hydrogel inhibited collagen deposition in the heart after I/R injury. Interstitial collagen deposition in the hearts was detected by Sirius Red staining (scale bar=100 μm). Data are expressed as mean ± SEM, n=5-8 animals per group. Significant differences were detected by one-way ANOVA with Tukey's multiple comparisons test, *p<0.05 , **p<0.01, ****p<0.0001.

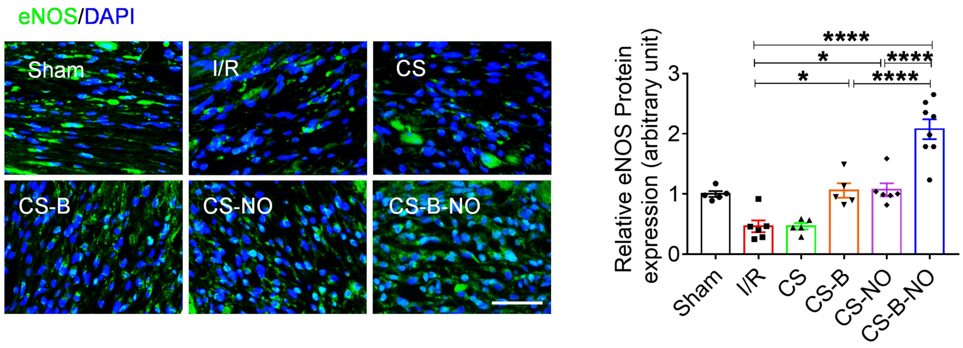

Figure S16. Representative images of eNOS immunostaining and quantification of the eNOS protein expression (scale bar=50 μm). Data are expressed as mean ± SEM, n=5-8 animals for each group. Significant differences were detected by one-way ANOVA with Tukey's multiple comparisons test, **p*<0.05 , *****p*<0.0001.

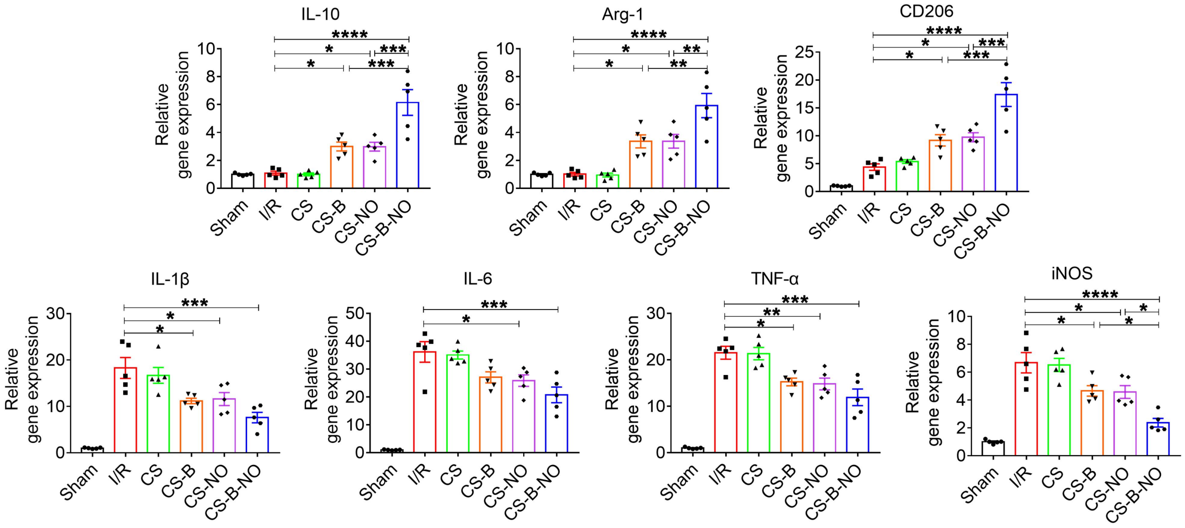

Figure S17. Effect of the CS-B-NO hydrogel on the expression of inflammatory-related genes. The expression of the inflammatory-related genes (IL-10, Arg-1, CD206, IL-1β, IL-6, TNF-α, iNOS) in the heart after I/R surgery was evaluated by RT-PCR. Data are expressed as mean ± SEM, n=5 individual experiments. Significant differences were detected by one-way ANOVA with Tukey's multiple comparisons test, *$p<0.05$, **$p<0.01$, ***$p<0.001$, ****$p<0.0001$.

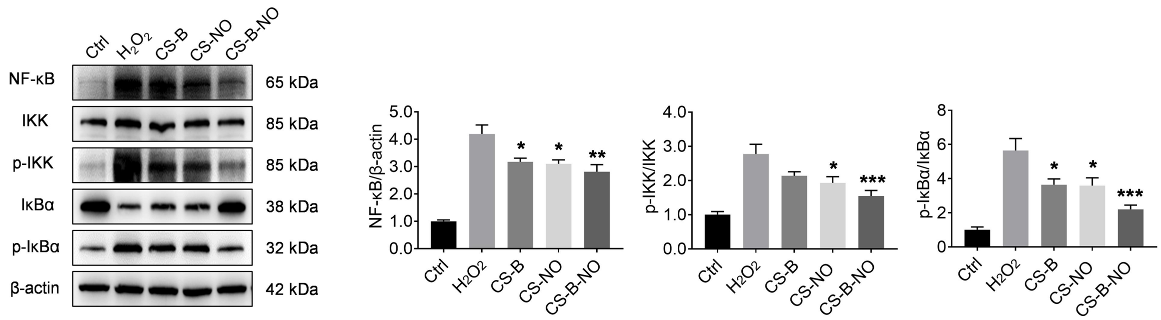

Figure S18. CS-B-NO hydrogel inhibited NF-κB signalling pathway through suppressing the phosphorylation of IKK/IκBα in H9C2 cells. Representative western blot images and related quantitative data showing the expression of NF-κB, IKK, p-IKK, IκBα, and p-IκBα in H9C2 cells. Data are expressed as mean ± SEM, n=5 individual experiments. Significant differences were detected by one-way ANOVA with Tukey's multiple comparisons test, *p<0.05 , **p<0.01, ***p<0.001.

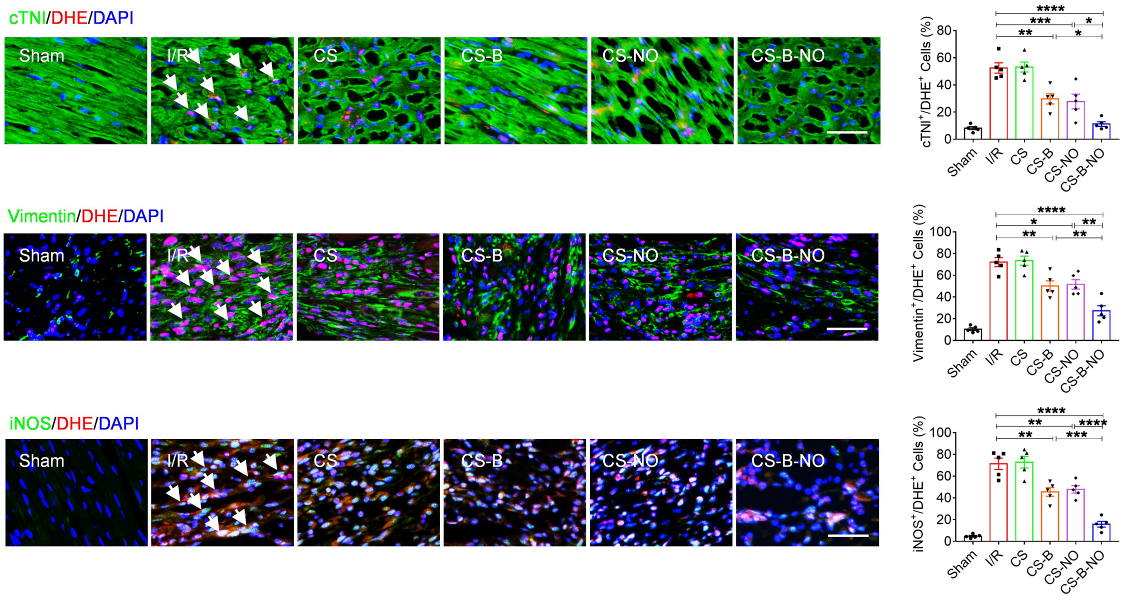

Figure S19. CS-B-NO hydrogel decreased ROS-positive cells in myocardium after I/R injury. Co-immunostaining was performed, and the number of cTNI^{+}/DHE^{+} myocardial cells, Vimentin $^{+}$/DHE^{+} fibroblasts and iNOS^{+}/DHE^{+} M1 macrophages in myocardial tissue at 1 day post-surgery was quantified, respectively (n=5 animals per group). Data are expressed as mean ± SEM. Significant differences were detected by one-way ANOVA with Tukey's multiple comparisons test, *p<0.05, **p<0.01, ***p<0.001, ****p<0.0001.

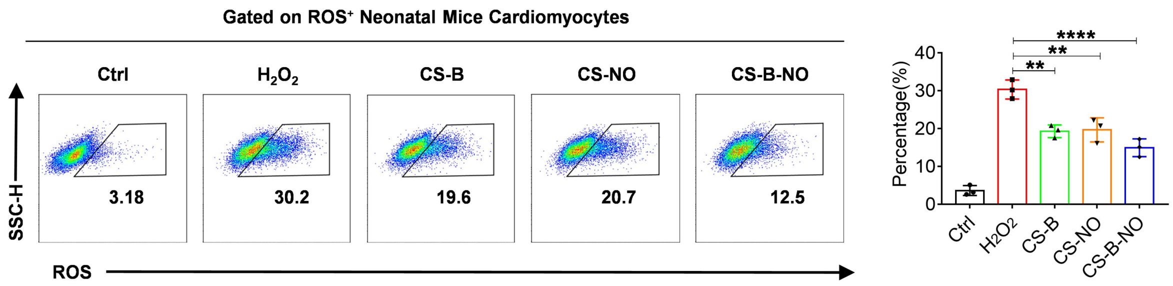

Figure S20. CS-B-NO hydrogel decreased ROS-positive neonatal mouse cardiomyocytes after H_2O_2 stimulation. Flow cytometry was performed to assess the number of ROS-positive neonatal mouse cardiomyocytes after H_2O_2 stimulation for 24 hours followed by different treatments. Data are expressed as mean ± SEM, n=3 individual experiments. Significant differences were detected by one-way ANOVA with Tukey's multiple comparisons test, $^{**}p<0.01$, $^{****}p<0.0001$.

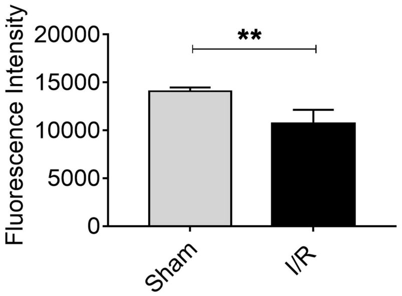

Figure S21. The free thiol content in heart tissues was determined by monobromobimane fluorescence assay. Data are expressed as mean ± SEM, n=5 animals for each group. Significant difference was detected by unpaired student t-test, $^{**}p<0.01$.

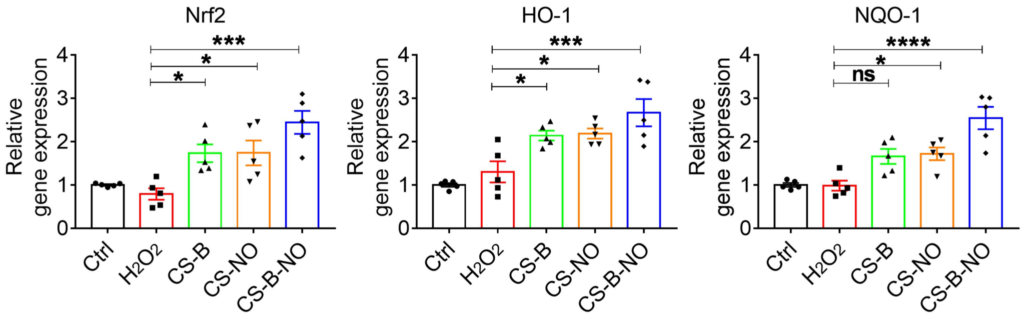

Figure S22. CS-B-NO hydrogel inhibited oxidative stress in H9C2 cells. The expression of the antioxidant-related genes (Nrf2, HO-1, NQO-1) in H9C2 cells after H_2O_2 stimulation was evaluated by RT-PCR. Data are expressed as mean ± SEM, n=5 individual experiments. Significant differences were detected by one-way ANOVA with Tukey's multiple comparisons test, **p*<0.05 , ****p*<0.001, *****p*<0.0001.

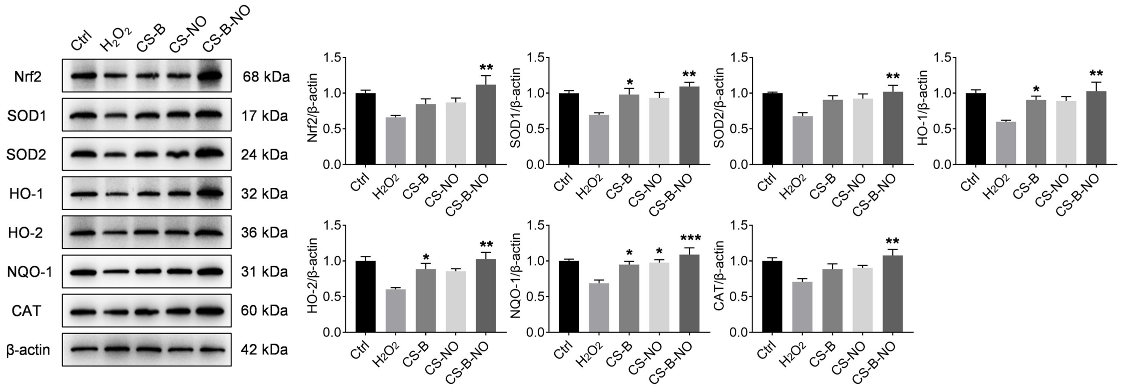

Figure S23. CS-B-NO hydrogel inhibited oxidative stress *in vitro*. The expression of the antioxidant-related proteins (Nrf2, SOD1, SOD2, HO-1, HO-2, NQO-1 and CAT) in H9C2 cells after H_2O_2 stimulation was evaluated by western blot. Data are expressed as mean ± SEM, n=5 individual experiments. Significant differences were detected by one-way ANOVA with Tukey's multiple comparisons test, **p*<0.05 , ***p*<0.01, ****p*<0.001.

References

[1] Q. Zhao, J. Zhang, L. Song, Q. Ji, Y. Yao, Y. Cui, J. Shen, P. G. Wang, D. Kong, *Biomaterials* **2013**, *34*, 8450.

[2] Y. Endo, T. Shirai, T. Akamatsu, K. Asada, *Ann. Allergy, Asthma, Immunol.* **2017**, *119*, 383.

[3] Y. Li, B. Chen, X. Yang, C. Zhang, Y. Jiao, P. Li, Y. Liu, Z. Li, B. Qiao, W. Bond Lau, X. L. Ma, J. Du, *Circulation* **2019**, *140*, 751.

[4] D. Zhu, J. Hou, M. Qian, D. Jin, T. Hao, Y. Pan, H. Wang, S. Wu, S. Liu, F. Wang, L. Wu, Y. Zhong, Z. Yang, Y. Che, J. Shen, D. Kong, M. Yin, Q. Zhao, *Nat. Commun.* **2021**, *12*, 4501.

[5] J. N. Simon, B. Vrellaku, S. Monterisi, S. M. Chu, N. Rawlings, O. Lomas, G. A. Marchal, D. Waithe, F. Syeda, P. R. Gajendragadkar, R. Jayaram, R. Sayeed, K. M. Channon, L. Fabritz, P. Swietach, M. Zaccolo, P. Eaton, B. Casadei, *Circulation* **2021**, *143*, 449.

[6] Q. Fan, R. Tao, H. Zhang, H. Xie, L. Lu, T. Wang, M. Su, J. Hu, Q. Zhang, Q. Chen, Y. Iwakura, W. Shen, R. Zhang, X. Yan, *Circulation* **2019**, *139*, 663.

[7] L. Xu, J. P. Eu, G. Meissner, J. S. Stamler, *Science (New York, N.Y.)* **1998**, *279*, 234.
